# Tailoring compatibility, morphology, and functional properties of polyester-based biodegradable blends: a review

**DOI:** 10.1039/d6ra07307g

**Published:** 2026-10-28

**Authors:** Vaishnu Suresh Kumar, Sarbani Ghosh, Mehran Ghasemlou, Banasri Roy, Benu Adhikari, Mohit Garg, Fugen Daver

**Affiliations:** a Department of Chemical Engineering, Birla Institute of Technology and Science (BITS) Pilani Campus, Vidya Vihar Pilani Rajasthan 333031 India mohit.garg@pilani.bits-pilani.ac.in; b Department of Chemical Engineering, The University of Melbourne Parkville Victoria 3010 Australia; c School of Science, RMIT University Bundoora Victoria 3083 Australia; d School of Engineering, RMIT University Bundoora Victoria 3083 Australia fugen.daver@rmit.edu.au

## Abstract

Understanding how thermodynamic compatibility, phase morphology, interfacial interactions, and processing conditions influence the performance and biodegradation of biodegradable polyester blends remains important and underreported. Thus, this review examines how compatibility and phase morphology govern the mechanical, barrier, and rheological properties and the underlying thermodynamics of PBS-, PBSA-, PHA-, and PBAT-based blends. The review starts by explaining the thermodynamic principles of polymer blends. An understanding of these principles is needed to interpret the immiscibility of biodegradable blends and its influence on phase behaviour, morphology, and functional properties. Thereafter, recent studies on polyester-based blends are discussed, focusing on the factors governing their mechanical, barrier, and rheological properties. Mechanical performance depends on the evolution of suitable phase morphology and interfacial adhesion which can facilitate stress transfer throughout the material. Reactive compatibilizers, interfacial crystallites, and nanostructures can improve toughness and strength by enhancing compatibility between the blend polymers. Barrier properties are governed by the degree of crystallinity, interface, and the tortuous diffusion pathways created by compatibilizers and nanoscale fillers. Rheological behaviour of the blends provides insight into molecular interactions, melt elasticity, and processability. In general, chain extension and filler-network formation improve melt strength and viscoelasticity. Excessive plasticization or phase separation tends to reduce melt elasticity and make processing more difficult. The effects of compatibilization, chain extension, and filler incorporation on biodegradation are non-linear. Thus, phase morphology, crystallinity, interfacial structure, and polymer accessibility need to modify and maintain the biodegradability of the blends. The insights from this review is essential for developing high-performance biodegradable polyester blends.

## Abstract 2

Linking thermodynamic understanding with morphology and compatibilization strategies is essential for designing biodegradable polyester blends that balance performance, processability, and end-of-life degradability.

## Graphical abstract

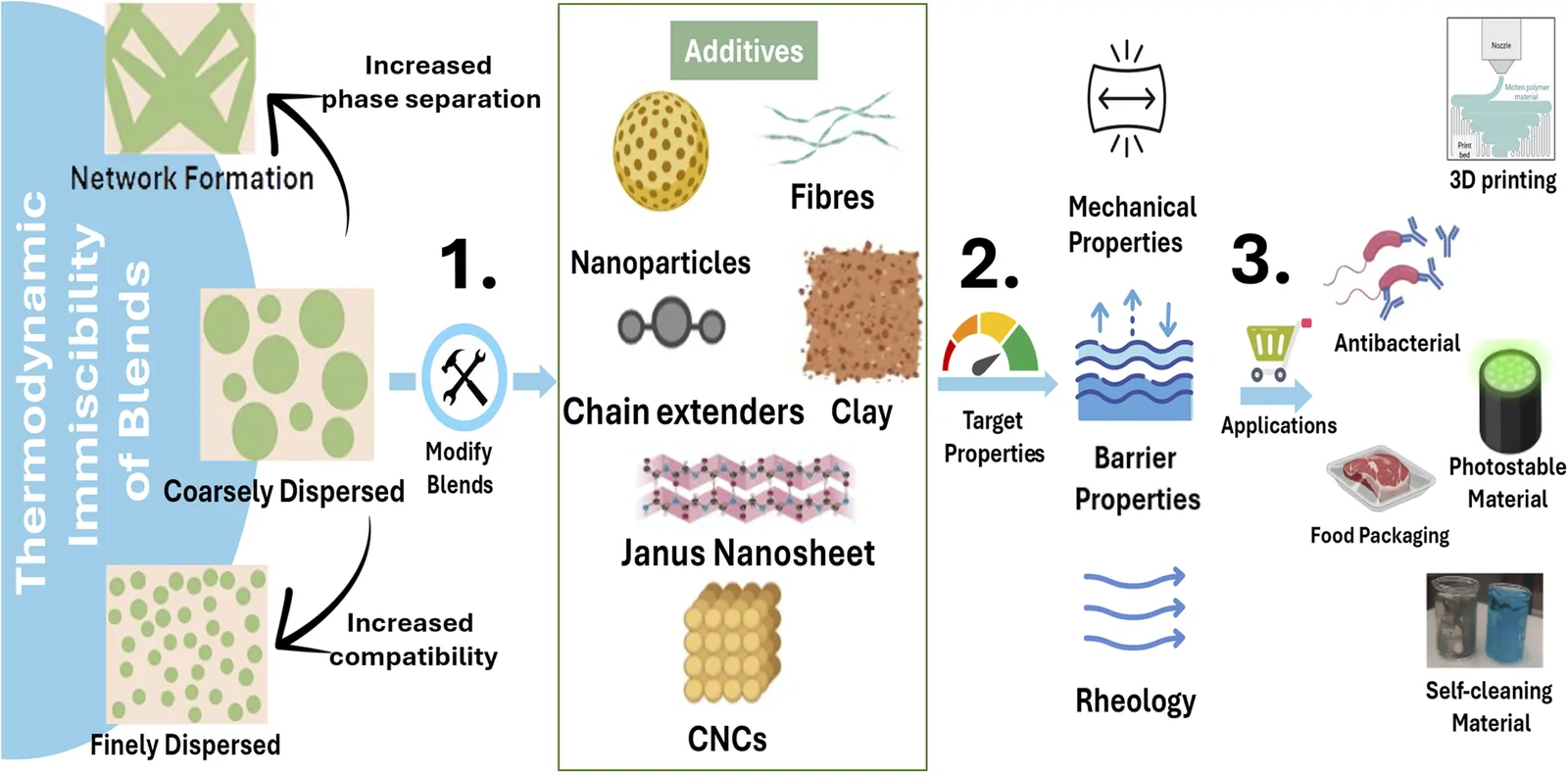

## 1 Introduction

Biodegradable polymers (biopolymers) are preferable over conventional non-biodegradable polymers for engineering materials, as they are eco-friendly, renewable, and biocompatible.^1^ Biopolymers, such as polylactic acid (PLA), starch, chitin, chitosan, poly(butylenesuccinate) (PBS), poly(hydroxyalkanoate) (PHA), and their derivatives/composites have been used in a variety of applications, including food packaging,^2^ insulating materials,^3^ textiles,^4^ medical,^5,6^ and pharmaceuticals.^7^ Some of these biopolymers even show functional properties that are comparable to conventional synthetic polymers.^8–10^ However, their wider adoption remains limited by higher cost and, in some cases, limited overall performance.^11–13^

PBS has emerged as a viable biodegradable polyester due to its balanced combination of flexibility, processability, and environmentally benign origin.^14,15^ PBS is synthesized from 1,4-butanediol and succinic acid, which can be derived from renewable resources. It exhibits a relatively low melting temperature (∼110 °C), an elastic modulus in the range of 200–700 MPa, and a degradation temperature around 250 °C, making it easier to process compared to many other biopolymers.^16^ Additionally, PBS possesses a low glass transition temperature (*T*_g_ ≈ −34 °C) and a crystallinity of up to ∼60%. These features make PBS ductile and thermally stable.^17^ PBS-derivative such as poly(butylene succinate-*co*-butylene adipate) (PBSA)^18^ have been developed to tailor its mechanical properties, processing characteristics, and degradation behaviour to increase its usage in wide range of applications. Poly(butylene adipate-*co*-terephthalate) (PBAT)^19^ is another important biodegradable polyester which has been widely used due to its excellent flexibility (% elongation at break ∼500–800%), mechanical performance (elastic modulus ∼50–200 MPa), and barrier properties (oxygen permeability ∼0.246 Barrer at 0% RH and 25 °C). Other widely studied polyester, such as PLA exhibit higher stiffness and strength, with an elastic modulus in the range of 2–4 GPa and melting temperatures between 140–200 °C (ref. 20) Similarly, polyhydroxyalkanoates (PHAs), including poly(3-hydroxybutyrate) (PHB),^21^ poly(3-hydroxybutyrate-*co*-3-hydroxyvalerate) (PHBV),^22^ and poly(3-hydroxybutyrate-*co*-3-hydroxyhexanoate) (PHBH),^23^ offer good mechanical strength and biodegradability, with melting temperatures around 170 °C and elastic moduli comparable to PLA.

Biodegradable polyesters are often used as blends or composites because their standalone properties may be insufficient for practical applications. Natural fibres,^24^ nanofillers,^25,26^ clays,^27^ essential oils,^28^ bio-based additives incorporated into biodegradable polyesters have proven to be effective in tailoring material performance. Such reinforcements modify the mechanical properties and also improve barrier performance by reducing gas permeability and increasing heat distortion temperature.^29^ Although these fillers and additives been widely used to alter the properties of resulting composite, each has certain drawbacks. Natural fibres such as nanocellulose, chitosan *etc.*, often show poor compatibility with polymer matrix and readily absorb moisture which reduce mechanical performance due to increase water sensitivity. Nanofillers can improve strength and barrier properties, but they tend to agglomerate, making it difficult to achieve uniform dispersion. Essential oils can provide antimicrobial and antioxidant functions, but their high volatility and low thermal stability hinders processability and storage.

In contrast to the traditional methods mentioned above, blending of two biopolymers is another cost-effective strategy to tailor the properties of the resulting polymer films. For example, PBS and its derivatives suffer from relatively low mechanical strength, which limits their standalone use in structural and packaging applications.^30^ To mitigate this, PBS is frequently combined with stiffer polymers, such as PLA and/or modified through crosslinking and compatibilization strategies to enhance its mechanical performance.^31^ In comparison, PLA exhibits high strength and stiffness but is inherently brittle and possesses poor barrier properties. This necessitates modifying its properties by blending it with ductile polymers like PBS or adding fillers to improve flexibility.^32^ Similarly, PHAs provide good mechanical strength but are limited by poor flexibility and thermal stability.^33^ PHAs also exhibit secondary crystallization at room temperature, which limits their long-term performance.^34^ The overall properties of biodegradable polyester blends can be tailored by optimizing the composition, dispersion, and interfacial interactions between the matrix and reinforcement.

In biopolymer blends, the selection of polymer pairs with complementary properties is essential to achieve a balanced combination of functional properties.^35^ The composition of individual polymers, their molecular weights, phase morphology, and interfacial adhesion play an crucial role in overall blend performance.^36^ The interface formed between the two polymer domains acts as the governing region that links structure to properties in immiscible biopolymer blends.^37,38^ Strong interfacial adhesion enables efficient stress transfer, which leads to improvements in strength and toughness. In contrast, low interfacial adhesion promotes debonding of the phases, cavitation induced rupture, and premature failure. Interfacial interactions also govern the viscoelastic properties of the blend. Compatibilization enhances complex viscosity and storage modulus.^39^ In contrast, incompatibility or excessive plasticization reduces melt strength and limits processability. The interfacial compatibility of the blends dictate the barrier properties of the blend by controlling the diffusion pathways of gas molecules. Increased interfacial interactions minimize voids and restrict the formation of easy diffusion pathways for gases and vapours. Additionally, morphologies such as core–shell structures and lamellar domains improve the barrier properties by creating torturous diffusion pathways for gas molecules. The gas diffusion further becomes difficult when nanofillers are uniformly dispersed and selectively localized at the interface.

The incompatibility of the biopolymers in the blends originate primarily from the unfavourable thermodynamics of polymer mixing.^37^ The high molecular weight, large polymer chains lower the entropy of mixing.^40^ Consequently, even chemically similar polyesters such as PBS, PBSA, PBAT, PLA, and PHAs often undergo phase separation during processing. The interplay of enthalpic interactions, molecular architecture, composition, and processing conditions govern the phase morphology, including dispersed, co-continuous, and interconnected microstructures.^41^ The morphology that develops as a result of thermodynamic interactions influences stress transfer, chain mobility, and diffusion through the material. These factors collectively determine the mechanical, rheological, and barrier properties of biodegradable blends. Therefore, understanding the thermodynamics of polymer mixing is important for designing high-performance biodegradable polymer blends with controlled morphology and interfacial properties.

Various compatibilization strategies have been developed to stabilize morphology and improve interfacial interactions in biopolymer blends to compensate for the thermodynamic immiscibility.^42,43^ This further governs the mechanical, viscoelastic, and barrier properties. Processing methods are also critical in determining the functional properties of biopolymer blends. Blending sequence, reactive extrusion, and masterbatch processing impact the filler dispersion and compatibilizer localization, thereby affecting morphology evolution and the resulting structure–property relationships.

Although numerous studies have investigated individual biodegradable polymers and their blends, a comprehensive understanding of how thermodynamic compatibility, phase morphology, interfacial interactions, and processing conditions collectively determine the mechanical, barrier, and rheological performance of PBS-, PBSA-, PBAT, and PHA-based systems remains important. The review summarizes recent studies (2021–2026) on biodegradable aliphatic polyesters such as PBS-, PBSA-, PHAs, and aliphatic–aromatic polyester PBAT-based biodegradable blends and composites, with particular emphasis on blend compatibility, phase morphology, interfacial interactions, and their relationships with mechanical, barrier, and rheological properties. Additionally, the thermodynamic basis of polymer immiscibility and phase behaviour is also discussed to establish the connection between molecular interactions and macroscopic performance. Finally, some emerging applications of these biodegradable blends as packaging, additive manufacturing, self-cleaning, and antimicrobial materials are highlighted. By integrating these aspects, this review aims to provide a structure–property framework for the rational design of high-performance biodegradable polyester blends.

## 2 Thermodynamic basis of phase behaviour and morphology in polymer blends

The thermodynamics of mixing plays a crucial role in the final phase morphology of the blends, which ultimately governs the mechanical, barrier, and rheological properties of the blends. The thermodynamics of mixing in binary blends depends on the Gibb's free energy of mixing (Δ*G*_mix_). The fundamental relation for Δ*G*_mix_ is given by eqn (1):^44^

1 Δ*G*_mix_ = Δ*H*_mix_ − *T*Δ*S*_mix_

A negative value of Δ*G*_mix_ indicates miscibility and a positive value indicates phase separation. Now, there are two contributions to Δ*G*_mix_, one is the enthalpy of mixing (Δ*H*_mix_) and other is the entropy of mixing (Δ*S*_mix_). While the enthalpic driving forces are dominant at lower temperatures, entropic driving forces play a crucial role at higher temperatures, *i.e.*, during melt processing.

The classical Flory–Huggins thermodynamic model provides important insight into the phase behaviour of polymer blends as a function of molecular weight and blend composition (volume fraction). The free energy of mixing is expressed by the following equation (eqn (2)):^40^2
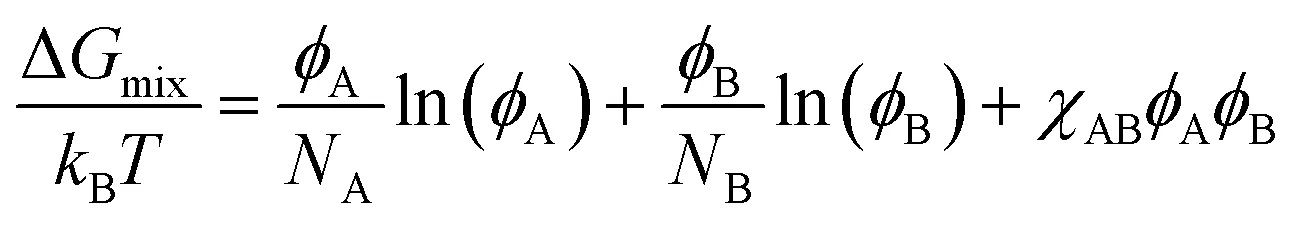
where, *ϕ*_A_ and *ϕ*_B_ are the volume fractions of polymer components A and B, respectively. *χ*_AB_ is the Flory–Huggins interaction parameter of mixing (or simply Flory parameter). *N*_A_ and *N*_B_ are chain length of two different polymers. The first two terms in the equation corresponds to Δ*S*_mix_ and the last term corresponds to Δ*H*_mix_. According to this equation, the extremely high molecular weight of polymer chains significantly reduces the entropy of mixing, causing most polymer pairs to exhibit miscibility or phase separation depending on volume fraction, and the Flory parameter.^40^ Consequently, even blends of chemically related aliphatic polyesters often exhibit phase-separated structures.^45^ Depending on composition and thermodynamic stability, biopolymer blends may undergo either spinodal decomposition, leading to co-continuous structures, or nucleation and growth, resulting in dispersed phase morphologies.^41^

Molecular dynamics simulations of PLA/PBS oligomeric chains provides the information about composition dependent phase miscibility by providing *χ*_AB_ interaction parameter. The study reveals that the Flory–Huggins parameter first decreases and then increases with increase in PBS content, thereby explaining the origin for phase behaviour in PLA/PBS blends.^46^

The classical Flory–Huggins framework has limitations when applied to real biodegradable polyester blends. The *χ*_AB_ interaction parameter is often treated as a simplified measure of polymer–polymer interactions, whereas real systems may have a complex interplay of interactions such as hydrogen bonding and dipole–dipole interactions with respect to the chemical structure of the polymers.^47^ In addition, the semicrystalline nature of many biodegradable polyesters introduces crystallization effects that are not explicitly captured by the classical mixing free-energy expression.^48^ Therefore, *χ*_AB_ interaction parameter should be considered an effective parameter rather than a universal measure of miscibility, and its interpretation should ideally be supported by complementary evidence such as morphology, thermal behaviour, crystallinity, and interfacial characteristics.

The classical Flory–Huggins model further fails to explain compressibility and free volume change upon mixing. As the morphological features are highly sensitive to processing conditions such as cooling rate and shear, and they play a critical role in determining end-use properties. The Equation of State (EOS) theories such as Sanchez–Lacombe,^49^ lattice fluid and cluster theory^48,49^ account for these complex interactions and are able to predict critical phenomenon such as Lower Critical Solution Temperature (LCST), which simpler incompressible models cannot typically capture.^50,51^ For example, in PLA/PBS blends, the equilibrium melting-point depression of PLA was used to derive Flory–Huggins interaction parameter which was supported by differential scanning calorimetry (DSC), wide-angle X-ray diffraction (WAXD), small-angle X-ray scattering (SAXS) and polarized optical microscopy.^52^

In addition to bulk phase behaviour interfacial thickness and adhesion plays important role in performance of biopolymers. These are governed by segmental interactions and chain interdiffusion. A diffuse interface can facilitate stress transfer and improve toughness, whereas a sharp interface may act as a site for mechanical failure.^53,54^

In an immiscible polymer blend, the formation of interface is associated with an excess free energy (eqn (3)).^55^

3 Δ*G*_interface_ = *γ*_12_*A*

where, *γ*_12_ is the surface tension between polymer phases 1 and 2 forming and interface, *A* is the interfacial area. The interfacial tension originates from the difference in molecular interactions between like and unlike polymer segments. When the interactions between the two polymers are less favourable than those within each individual phase, the formation of an interface becomes energetically unfavourable (higher Δ*G*_interface_). Consequently, the thermodynamics of an immiscible blend is influenced by both the free energy of mixing and the energetic penalty associated with forming the inter-polymer interface. The magnitude of this interfacial energy therefore provides an important thermodynamic measure of the compatibility between the phases and influences the extent of phase separation and the stability of the resulting interface. The tendency to minimize interfacial free energy can therefore influence the size, shape, and distribution of the dispersed phase, while changes in blend composition may further promote the transition from discrete domains to more interconnected morphologies. These interfacial characteristics further influence diffusion pathways for gases and vapours, thereby affecting barrier properties, as well as chain mobility under deformation, which governs rheological behaviour.^56^ In a PLA/PBAT/PBS ternary blend with a fixed PLA : PBAT ratio, the high *χ*_AB_ interaction parameter between PLA and PBAT, compared with the lower values for PLA/PBS and PBS/PBAT, suggests that PBS preferentially accumulates at the PLA/PBAT interface.^57^ This is further confirmed by polarized optical microscopy.

While eqn (3) describes the macroscopic thermodynamic cost of forming an interface, molecular and continuum approaches such as the Helfand–Tagami model^58^ and Cahn–Hilliard theory,^59^ respectively, provide further insight into the development of phase morphology and the molecular structure and energetics of the polymer–polymer interface.

The Helfand–Tagami theory provides a molecular-level description of the interface between two immiscible polymer phases by considering the balance between the unfavourable interactions between unlike polymer segments and the conformational entropy associated with polymer chains near the interface. Within this framework, the interfacial width decreases with increasing *χ*_AB_ interaction parameter, approximately following 
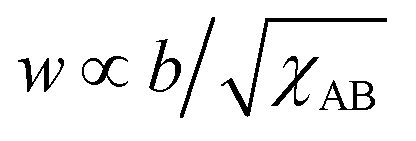
, while the interfacial tension increases with the strength of unfavorable segmental interactions. Thus, increasing *χ* produces a sharper and energetically more costly polymer–polymer interface. In contrast to bulk thermodynamic models, the theory describes the spatial variation of polymer concentration across the interface and relates the resulting interfacial structure to the Flory–Huggins interaction parameter, polymer chain length, and segment dimensions. A higher *χ*_AB_ interaction parameter generally corresponds to stronger segregation, a sharper concentration gradient, and higher interfacial tension. Unlike that, weaker unfavourable interactions allow greater chain interpenetration and a broader interface.

The Cahn–Hilliard model describes the spatial evolution of composition during phase separation. It considers both the bulk free energy of mixing and the energetic penalty associated with composition gradients:4
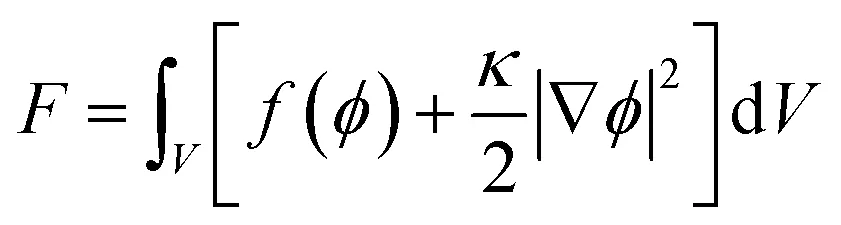
where *f*(*ϕ*) is the homogeneous free-energy density, *ϕ* is the local composition, and the gradient term represents the energetic cost of creating an interface. Thus, Cahn–Hilliard can describe the spatial evolution of phase separation and the development of phase-separated morphologies.^60^

In multiphase polymer blends, the incorporation of a filler introduces additional interfaces and therefore adds another level of complexity to the phase morphology. The filler may preferentially reside within one polymer phase, distribute between the phases, or localize at the polymer–polymer interface, depending on its interactions with the constituent polymers. Such localization can modify the phase morphology, interfacial area, and stress transfer, and consequently influence the functional properties of the blend. The preferred localization is therefore governed not only by the properties of the filler itself but also by the relative thermodynamic affinities of the filler toward the two polymer phases. The selective localization of a filler in a polymer blend can be described using the balance of interpolymer and polymer–filler interfacial energies. For a binary polymer blend A/B containing a filler *F*, the interfacial tensions are *γ*_AB_, *γ*_AF_, and *γ*_BF_. The tendency of the filler to preferentially localize within one polymer phase or at the polymer–polymer interface can be described using the wetting coefficient:^61^5
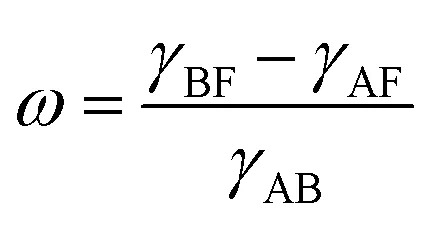


The relative magnitude of these interfacial energies determines the preferred localization of the filler. Interfacial localization is favoured when it provides a lower free-energy configuration than localization within either bulk phase. Thus, filler surface chemistry and its affinity toward the constituent polymers can influence filler distribution, phase separation, domain size, and interfacial area, providing a thermodynamic basis for linking filler-induced morphological changes with the mechanical properties of the blend. In PLA/PBAT blends containing cellulose nanocrystals (CNCs), surface-energy-derived interfacial tensions were used to calculate a wetting coefficient of 6.67, predicting equilibrium localization of CNCs in the PBAT phase.^62^

The thermodynamics of polymer blends provide the basis for understanding how compatibilization, chain extension, processing, and filler localization modify blend morphology. The resulting changes in domain size, phase connectivity and interfacial characteristics ultimately influence the mechanical, barrier, and rheological performance of the material. The impact of phase behaviour and their resulting influence on mechanical and rheological properties of biopolymer blends and composites will be discussed in the next sections.

## 3 Mechanical properties

Mechanical properties determine the structural integrity, durability, and practical applicability of polymeric materials, particularly in packaging and biomedical applications.^37,63^ They are generally evaluated using parameters, such as elastic modulus (EM), tensile strength (TS), elongation at break (% EAB), and impact strength (IS). These parameters describe the stiffness, strength, ductility, and energy absorption of a material, respectively.

Mechanical performance of biodegradable polymer blends depends on both the intrinsic molecular characteristics of the constituent polymers and structural features developed during blending such as phase morphology and interfacial interactions.^64,65^ Chain flexibility, molecular weight, crystallinity, and glass transition temperature are among the key factors influencing their mechanical response. However, individual polymers may not provide the balance of mechanical properties required for specific applications. Several strategies have been explored to tailor the mechanical performance of biodegradable polymer blends, including polymer blending, compatibilization, filler incorporation, chain extension, and interfacial engineering.^63,66–68^ Processing conditions can also influence the resulting mechanical properties.^63^ These approaches modify the composition, morphology, and interfacial interactions within the blend and can therefore alter its mechanical response. The effectiveness of each strategy depends on the polymer system and the conditions used. Therefore, careful control of these factors is necessary to achieve the desired balance of stiffness, strength, and toughness for specific applications. Building on these principles, recent studies have focused on tailoring composition, morphology, and interfacial interactions in biodegradable polyester-based systems to improve their overall mechanical performance. The following sections highlight these approaches to mechanical reinforcement, their underlying mechanisms, and the associated challenges.

### 3.1 Plasticizing and toughening by a soft phase

Blending a stiff biopolymer (*i.e.* higher EM) with a softer biopolymer (*i.e.* lower EM) is a common strategy to modify the mechanical properties of the biopolymeric material. Softer polymers generally lower the modulus of the blend, act as a plasticizer to increase the ductility of the blend system. For example, PBS can plasticize the polymers such as PHB,^69^ which decreases its EM from 2772.08 MPa for neat PHB to 2047.61 MPa in the 50% PBS blend with corresponding increase in % EAB and IS, demonstrating improved ductility and toughness of the blends (Table 1, S. no. 1). This behaviour is associated with the increased chain mobility introduced by the softer PBS phase, which facilitates plastic deformation and energy dissipation. A comparable trend was observed for the poly(3-hydroxybutyrate-*co*-3-hydroxyvalerate) PHBV/PBS blends, where the incorporation of PBS reduced stiffness and enhanced ductility and impact resistance (Table 1, S. no. 2). Similarly, PBS, poly(butylene adipate-*co*-terephthalate) (PBAT), and thermoplastic starch (TPS) has a plasticizing impact on PLA. TPS, PBS, and PBAT produced different reductions in the modulus of PLA despite all acting as soft phases, reflecting differences in their intrinsic flexibility and interactions with the PLA matrix.^70^ The EM of PLA decreased substantially from 3540 MPa for neat PLA to 620 MPa in the blend containing 70% TPS. In comparison, the EM of PLA decreased to 1270 MPa and 970 MPa in blends containing 50% PBS and 70% PBAT, respectively, indicating a relatively weaker plasticizing effect than TPS (Table 1, S. no. 4). The plasticizing effect of a polymer phase depends on not only on its intrinsic stiffness, but also on its chain mobility, molecular structure, crystallinity, and interactions with the matrix. These factors determine how effectively the soft phase facilitates deformation of the blend.

**Table 1:** Summary of the mechanical properties of biodegradable polyester blends

S. No.	Biopolymer	Dispersed phase(s)	Ratio (biopolymer : dispersed phase)	Youngs modulus (MPa)	Tensile strength (MPa)	Impact strength (J m^−1^)	% EAB	Reference
**Uncompatibilized binary blends**								
1	PHB	Bio-PBS	100 : 00 : 00	2772.08	40.16	3.43	1.44	69
			90 : 10 : 00	2641.96	37.91	3.87	1.57	
			70 : 30 : 00	2195.25	27.67	4.16	1.14	
			50 : 50 : 00	2047.61	32.82	5.38	1.81	
			00 : 100 : 00	721.67	34.19	17.33	10.02	
2	PHBV		100 : 00 : 00	2906.48	37.14	3.6	1.43	
			90 : 10 : 00	2484	30.66	4.04	1.3	
			70 : 30 : 00	2254	30.93	4.2	1.6	
			50 : 50 : 00	1780.48	28.49	4.28	2.2	
			00 : 100 : 00	721.67	34.19	17.33	10.02	
3	PBS	(Acrylonitrile-butadiene-styrene) ABS	100 : 00 : 00	2.23	0.16	—	2.23	90
			90 : 10 : 00	2.6	0.23	—	2.6	
			80 : 20 : 00	3.97	0.37	—	3.97	
			70 : 30 : 00	4.43	46	—	4.43	
4	PLA	TPS	70 : 30 : 00	2260	36	—	8	70
	PLA	PBAT	70 : 30 : 00	2490	38	—	6	
	PBS	PBAT	70 : 30 : 00	253.49	26.99	—	—	

**Fibre-reinforced biodegradable composites**								
5	Bio-PBS	Hemp fiber	100 : 00 : 00	—	26	85	—	91
			90 : 10 : 00	—	28	40	—	
			80 : 20 : 00	—	32	52	—	
			70 : 30 : 00	—	34	74	—	
6	PBS-OL (with overlap)	—	—	530	36.5	—	—	85
	PBS	Short fibre (SF) hemp	5% hemp	820	27	—	—	
	PBS-OL	SF-hemp	5% hemp	850	28.5	—	—	
	PBS-OL-HT (high temperature)	—	—	880	27.5	—	—	
	PBS	LF (long fibre)-hemp	5% hemp	840	24	—	—	
	PBS-OL	LF-hemp	5% hemp	890	26.5	—	—	
	PBS-OL-HT	LF-hemp	5% hemp	880	27	—	—	

**Reactive compatibilizers and chain extenders**								
7	PBS	—	100 : 00 : 00	195	—	—	440	92
	PBS	Ester compatibilizer (GMA-based)	95 : 05 : 00	218	—	—	676	
	PBS	Clay (D43B)	97 : 03 : 00	319	—	—	54	
	PBS	Ester compatibilizer (GMA-based)/clay (D43B)	92 : 05 : 03	228	—	—	364	
	PBS	PLA	70 : 30 : 00	540	—	—	247	
	PBS	PLA/ester compatibilizer (GMA-based)	70 : 05 : 25	559	—	—	267	
8	CS/PLA-ADR	PBS	95 : 05 : 00	690	68.5	27.5	17.5	34
	CS/PLA-ADR	PBS	90 : 10 : 00	780	63.5	29.5	100	
	CS/PLA-ADR	PBS	85 : 15 : 00	630	59.5	35	115	
	CS/PLA-ADR	PBS	80 : 20 : 00	610	56	46.5	158	
	CS/PLA-ADR	PBS	75 : 25 : 00	700	54.5	24	125	
	CS/PLA-ADR	PBS	70 : 30 : 00	570	48.5	26	60	
9	PLA/PBAT	Joncryl (150 °C)	(75 : 25) : 5	1125	45	—	55	86
	PLA/PBAT	Joncryl (170 °C)	(75 : 25) : 5	1155	43.5	—	30	
	PLA/PBAT	Joncryl (190 °C)	(75 : 25) : 5	1145	42.5	—	35	
	PLA/PBAT	Joncryl (210 °C)	(75 : 25) : 5	1135	40.5	—	18	
	PLA/PBAT	(150 °C)	(75 : 25)	1105	38.5	—	30	
	PLA/PBAT	(170 °C)	(75 : 25)	1120	36	—	22	
	PLA/PBAT	(190 °C)	(75 : 25)	1090	37	—	24	
	PLA/PBAT	(210 °C)	(75 : 25)	1065	35.5	—	20	
10	PLA	—	—	—	70.6	—	16.1	83
	PLA	PBAT	70 : 30 : 00	—	36.5	—	328.2	
	PLA/PBAT	DCP	(69.8 : 29.91) : 0.30	—	42.2	—	369.6	
	PLA/PBAT	MG	(69.31 : 29.70) : 0.99	—	52.4	—	409.7	
	PLA/PBAT	MG	(63.64 : 27.27) : 9.09	—	58.8	—	366.4	
11	PLA	PBAT	70 : 30 : 00	—	43.4	9600	93	76
	PLA/PBAT	PLA-*g*-ECA	67 : 30 : 03	—	42.2	11 800	117	
	PLA/PBAT	PLA-*g*-ECA	64 : 30 : 06	—	41.9	12 400	153	
	PLA/PBAT	PLA-*g*-ECA	60 : 30 : 10	—	41.6	12 500	251	
	PLA/PBAT	PLA-*g*-ECA	55 : 30 : 15	—	41.2	12 100	159	
12	PLA/PBAT	MA-*g*-PBAT	89 : 10 : 01	1869.5	—	—	29.9	80
	PLA/PBAT	MA-*g*-PBAT	87 : 10 : 03	1879.1	—	—	22.7	
	PLA/PBAT	MA-*g*-PBAT	85 : 10 : 05	1709	—	—	68.7	
	PLA/PBAT	MA-*g*-PBAT	82 : 10 : 08	1785	—	—	8	
	PLA/PBAT	MA-*g*-PBAT (high molecular weight)	89 : 10 : 01	1882	—	—	44.4	
	PLA/PBAT	MA-*g*-PBAT (high molecular weight)	90 : 10 : 01	1659.1	—	—	233.7	
	PLA/PBAT	MA-*g*-PBAT (high molecular weight)	91 : 10 : 01	1662.6	—	—	151	
	PLA/PBAT	MA-*g*-PBAT (high molecular weight)	92 : 10 : 01	1656.6	—	—	16.9	

**Nanofiller-modified blends and nanocomposites**								
13	PBS	PLA/clay (D43B)	70 : 30(97PLA : 3D43B)	1020	—	—	131	93
	PBS/ester compatibilizer (GMA-based)	PLA/clay (D43B)	75(95PBS : 5GMA) : 25(97PLA : 3D43B)	617	—	—	335	
	PBS	PLA/ester compatibilizer (GMA-based)/clay (D43B)	70 : 22 : 5 : 3	—	—	—	—	
14	PBS-PBAT	Lignin–ZnO	(70 : 30) : (95 : 5)	261.88	26.05	—	—	84
	PBS-PBAT	Lignin–ZnO	(70 : 30) : (90 : 10)	304.52	24.03	—	—	
	PBS-PBAT	Lignin–ZnO	(70 : 30) : (85 : 15)	347.44	22.57	—	—	
15	PLA/PBAT	—	—	—	21	—	30	73
	PLA/PBAT	Janus nanosheet (JN)	0.1 JN	—	24	—	55	
	PLA/PBAT	Janus nanosheet (JN)	0.3 JN	—	28	—	110	
	PLA/PBAT	Janus nanosheet (JN)	0.5 JN	—	23	—	85	
	PLA/PBAT	Janus nanosheet (JN)	1 JN	—	22	—	45	

**Ternary blends and interface-engineered systems**								
16	PLA/PGA/PBS	—	50 : 10 : 40	—	41	—	5	82
	PLA/PGA/PBS	—	60 : 10 : 30	—	36	—	4	
	PLA/PGA/PBS	—	70 : 10 : 20	—	46	—	11.5	
	PLA/PGA/PBS	—	80 : 10 : 10	—	43	—	7.5	
	PLA/PGA/PBS	ADR	60 : 10 : 40 (1 phr ADR)	—	43	—	5.5	
	PLA/PGA/PBS	ESO3-ECD	60 : 10 : 40 (1 phr ESO3-ECD)	—	47	—	20	

Zhao *et al.*^71^ also demonstrated the plasticizing effect of PBS on PLA. Increasing the PBS content from 10% to 50% in the PLA/PBS blend increased the % EAB of the blend from 13.2% to 16.1% and IS from 5.1 to 8.3 KJ m^−2^. The EM of the blend, however, decreased from 1980 to 486 MPa at higher PBS content. At higher PBS contents, the lower miscibility between PLA and PBS can lead to weaker interfacial adhesion and poor stress transfer between the two phases. The miscibility of polymer blends is governed by the Gibbs free energy of mixing (eqn (2)), where both the enthalpic interaction between the polymer segments and the entropy of mixing contribute to the overall free energy. Because of their high molecular weights, the entropy gain upon mixing of PLA and PBS is relatively small. Therefore, even modest unfavourable segmental interactions, represented by a positive *χ* parameter, can result in a positive free energy of mixing and promote phase separation. At higher PBS contents, the lower thermodynamic affinity between PLA and PBS can therefore lead to increased phase separation and weaker interfacial adhesion. Under tensile loading, this weak interface can promote cavitation and interfacial debonding, which allows greater deformation and contributes to the increase in % EAB and IS. However, the resulting loss of interfacial load transfer reduces the ability of the PLA matrix to effectively transmit stress to the PBS phase, leading to a pronounced decrease in EM.

These observations illustrates the fundamental trade-off which often arises when a stiff biodegradable polymer is blended with a softer phase. Increasing the concentration of soft-phase content promotes chain mobility and deformation, improving ductility and potentially toughness, but simultaneously reduces stiffness and depending on the interfacial adhesion and morphology, it may also reduce tensile strength. Poor interfacial compatibility can further reinforce this trade-off by limiting stress transfer and causing interfacial debonding. Consequently, simple blending may shift the material from a stiff and brittle response towards a more ductile one without necessarily improving its overall mechanical performance. Achieving a balanced combination of stiffness, strength, and toughness therefore requires control over both phase morphology and interfacial interactions.

### 3.2 Morphology refinement

Once a soft phase is introduced, how can its size, distribution, and connectivity be controlled to balance stiffness, strength, and toughness. Simple blending often involves a trade-off between the increased ductility provided by the soft phase and the loss of stiffness and strength. This trade-off highlights the importance of controlling phase morphology and interfacial compatibility. In particular, the size and distribution of the dispersed phase determine the interfacial area and phase connectivity, which in turn influence stress transfer and the overall mechanical response of the blend. Righetti *et al.*^72^ demonstrated this relationship by correlating the mechanical properties and the morphology of PBS/PBSA phases in their blend with PHB. SEM analysis (Fig. 1(a)) revealed that at low concentrations of the second phase (15/85 and 85/15 compositions), in both PHB/PBS and PHB/PBSA blends showed a typical matrix–droplet morphology with uniformly dispersed domains of approximately 0.5 µm. Once phase separation occurs, the interfacial free energy further influences the morphology, with the system tending to minimize the interfacial area by forming dispersed droplets. As the amount of the dispersed phase increased, the droplet size also increased (especially in the PHB/PBSA blends). In contrast, the PHB/PBS blends showed less obvious phase separation at intermediate compositions and gradually developed co-continuous morphologies over a wider composition range than the PHB/PBSA systems. PBS/PBSA polymers at lower proportions form dispersed droplets within PHB because this morphology reduces the interfacial area relative to highly interconnected structures and reduce the energetic penalty to form the interface (eqn (3)). When PBS/PBSA concentration increases, droplets form closer contacts with each other and can deform, collide, and eventually interconnect. The 50/50 and 30/70 blends clearly exhibited interconnected co-continuous structures. These morphological changes modified the mechanical response of the blends. The formation of co-continuous structures improved phase connectivity and allowed more efficient stress transfer between the phases, leading to higher ductility and the appearance of necking during deformation. As a result, the blends gradually changed from brittle to ductile behaviour with increasing PBS or PBSA content, accompanied by higher % EAB values. Although the EM decreased with increasing PBS/PBSA content because of the softer nature of these phases (Fig. 1(b) and (c)), the values remained relatively stable and followed the rule of mixtures, suggesting effective load sharing between the phases. Despite the thermodynamic immiscibility of the blends, this behaviour was due to strong physical adhesion caused by crystallization-induced shrinkage which improved interfacial contact and stress transfer.

**Fig. 1:**
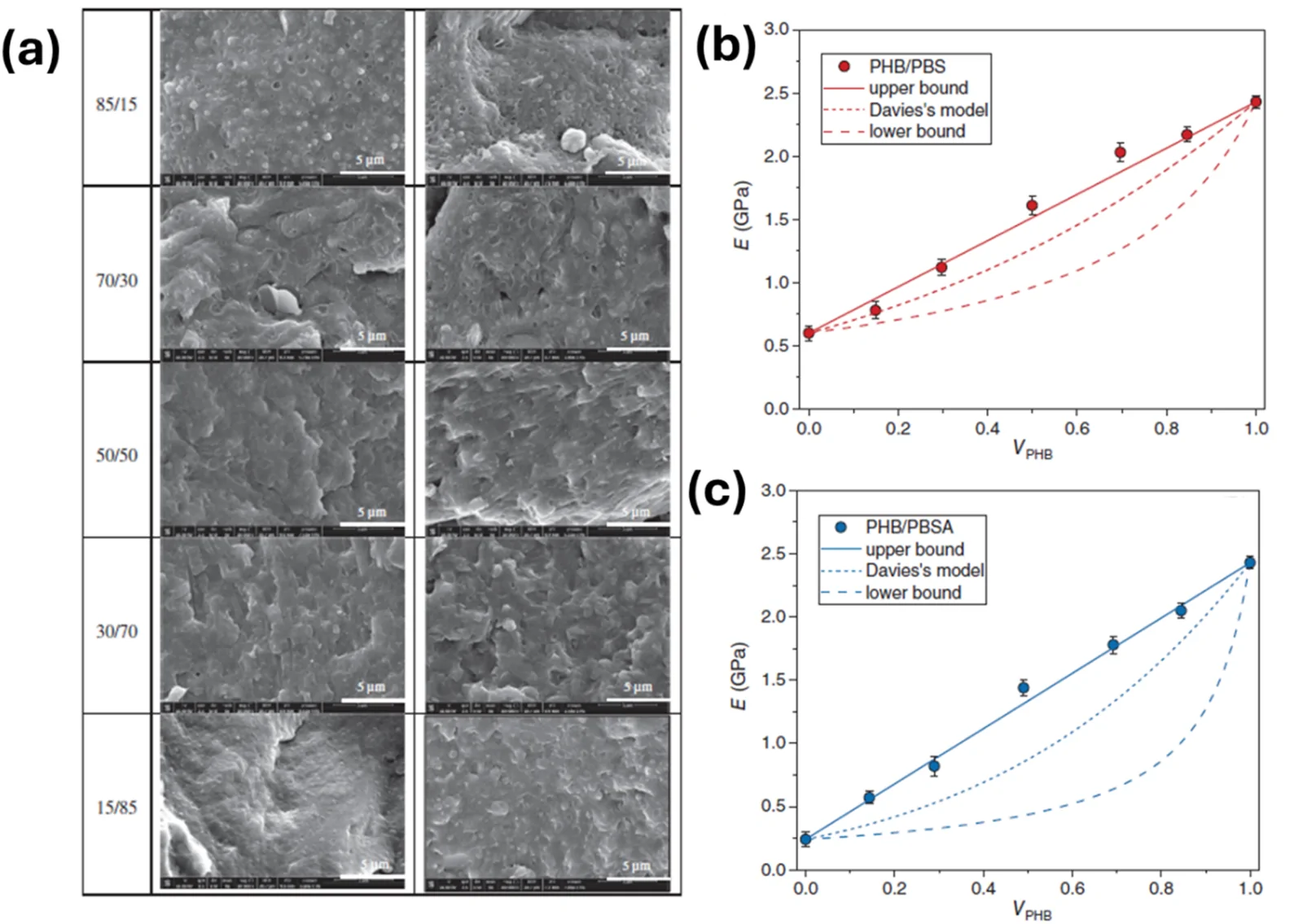
(a) SEM micrographs of PHB/PBS (left column) and PHB/PBSA (right column) blends at ×20 000 magnification. Variation of elastic modulus with PHB volume fraction for (b) PHB/PBS and (c) PHB/PBSA blends. The solid, dashed, and dotted lines represent the elastic modulus predicted by the parallel (upper bound), series (lower bound), and Davies models, respectively. Reproduced under the Creative Commons Attribution (CC BY) License from ref. 72. Copyright 2021. John Wiley Sons Ltd.

Ternary biodegradable blends involve more complex interfacial interactions than binary blends, making morphology refinement and compatibilization particularly important for achieving balanced mechanical properties. The reactive and non-reactive ternary blends of PLA, PBS, and PBAT show different morphology evolution and resulting mechanical properties. In non-reactive PLA/PBAT/PBS blends, PBS acted as a physical compatibilizer by localizing at the interface and forming micro-spherulites, which reduced interfacial tension and refined the morphology from coarse dispersed domains to finer micro-aggregates. PBS as a compatibilizer reduces the interfacial free energy between the polymer phases. This lowering of interfacial energy results in a finer and more stable morphology that improves stress transfer and ductility. This morphological improvement enhanced ductility, with % EAB increasing from ∼70% to ∼300%, and moderately improved TS from ∼19 MPa to ∼28 MPa, while maintaining a relatively constant EM. In contrast, reactive blends exhibited further enhancement in interfacial compatibility due to the action of peroxide and carbodiimide agents, which promoted chain extension and possible crosslinking between phases. This led to a more stabilized and finer morphology with stronger interfacial adhesion, enabling efficient stress transfer. As a result, reactive blends maintained tensile strength at ∼30 MPa and exhibited high ductility (% EAB 250%). However, a slight reduction in elongation was observed at higher PBS contents due to increased network rigidity. Thus, reactive compatibilization produced a more stable and refined morphology, leading to improved and more consistent mechanical performance.^57^

Controlling phase morphology through interfacial localization is also an effective strategy to improve the mechanical performance of biopolymer blends. Zhang *et al.*^73^ used anisotropic Janus nanosheets (JNs) to refine the phase morphology of PLA/PBS blends. The incorporation of silica-based JNs refined the phase morphology and enhanced interfacial adhesion, resulting in improved mechanical performance. In the absence of JNs, the blends exhibited brittle behaviour due to poor compatibility between PLA and PBS. However, the amphiphilic nature of JNs enabled their preferential localization at the interface (Fig. 2), where they formed a dense interfacial layer that strengthened interfacial bonding and improved stress transfer. As a result, both strength and ductility improved significantly, with TS increasing by up to ∼37% and % EAB increasing by up to ∼224% at ∼0.3 wt% JN content (Table 1, S. no. 15). Morphological analysis showed smaller PBS domains and less distinct phase boundaries, indicating better dispersion and improved interfacial adhesion. However, increasing the JN loading beyond 0.3 wt% caused excessive nanosheet accumulation at the interface, leading to interfacial debonding and reduced mechanical performance.

**Fig. 2:**
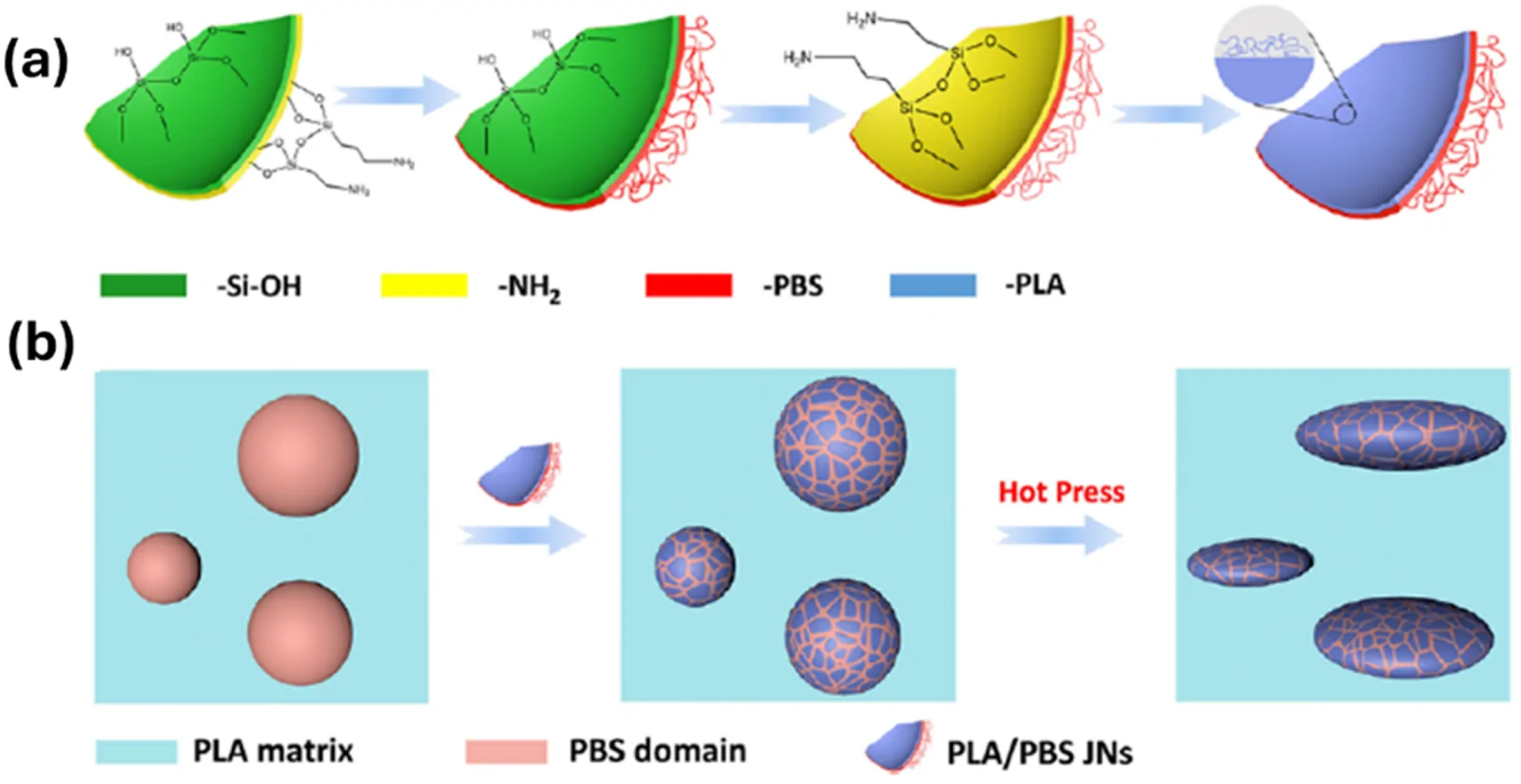
(a) Schematic illustration of the synthesis of PLA/PBS Janus nanosheets (JNs) *via* successive grafting of PBS chains onto the amine-functionalized side and PLA chains onto the silanol-functionalized side; (b) interfacial stabilization of PLA/PBS blends by PLA/PBS JNs acting as solid compatibilizers, along with the formation of lamellar PBS domains after hot pressing. Reproduced with permission from ref. 73. Copyright 2022. American Chemical Society.

While JNs refine the phase morphology through selective interfacial localization, interfacial crystallization provides another route for refining the domain size and generating a reinforcing structure. Wang *et al.*^74^ demonstrated this approach by constructing interfacial stereocomplex (SC) crystallites of poly-d-lactic acid (PDLA) at the interface of poly-l-lactic acid (PLLA)/PBAT blends (Fig. 3(a–c)). The formation of SC crystallites at the interface effectively enhanced the mechanical performance of (PLLA)/PBAT blends by simultaneously improving interfacial compatibility and crystallization behaviour. The thermodynamic immiscibility of PLLA and PBAT drives phase separation during processing. The formation of interfacial SC crystallites reduced the interfacial free energy and stabilized a finer morphology while promoting crystallization. The improved interfacial adhesion and stress transfer, resulted in higher toughness without sacrificing strength. Reactive blending with ESA-*graft*-PDLA led to the *in situ* formation of SC crystallites through co-crystallization between PDLA grafts and PLLA. These interfacial SC crystallites preferentially located at the phase interface. Crystallite significantly refined the morphology and reduced PBAT domain size to ∼0.4–0.8 µm, improving interfacial compatibility and strengthening interfacial adhesion and facilitating efficient stress transfer. As a result, the toughness increased substantially, with IS rising from 3.6 kJ m^−2^ for the uncompatibilized PLLA/PBAT (70/30) blend to 39.6 kJ m^−2^ at 0.5 wt% ESA-*graft*-PDLA, reaching 53.2 kJ m^−2^ at the optimal loading. Unlike conventional compatibilization approaches, SC crystallites also acted as highly efficient nucleating agents. The SC crystallites increased crystallinity from ∼15% to ∼39–50%, which contributed to maintaining TS (∼48.1 MPa) and modulus while significantly enhancing heat resistance (HDT ∼124 °C). They also improved interfacial adhesion and promoted crystallization, resulting in high toughness while maintaining strength and thermal stability. However, beyond the optimal loading (∼1.5 wt%), the improvement in toughness became marginal, indicating saturation of the interfacial sites.

**Fig. 3:**
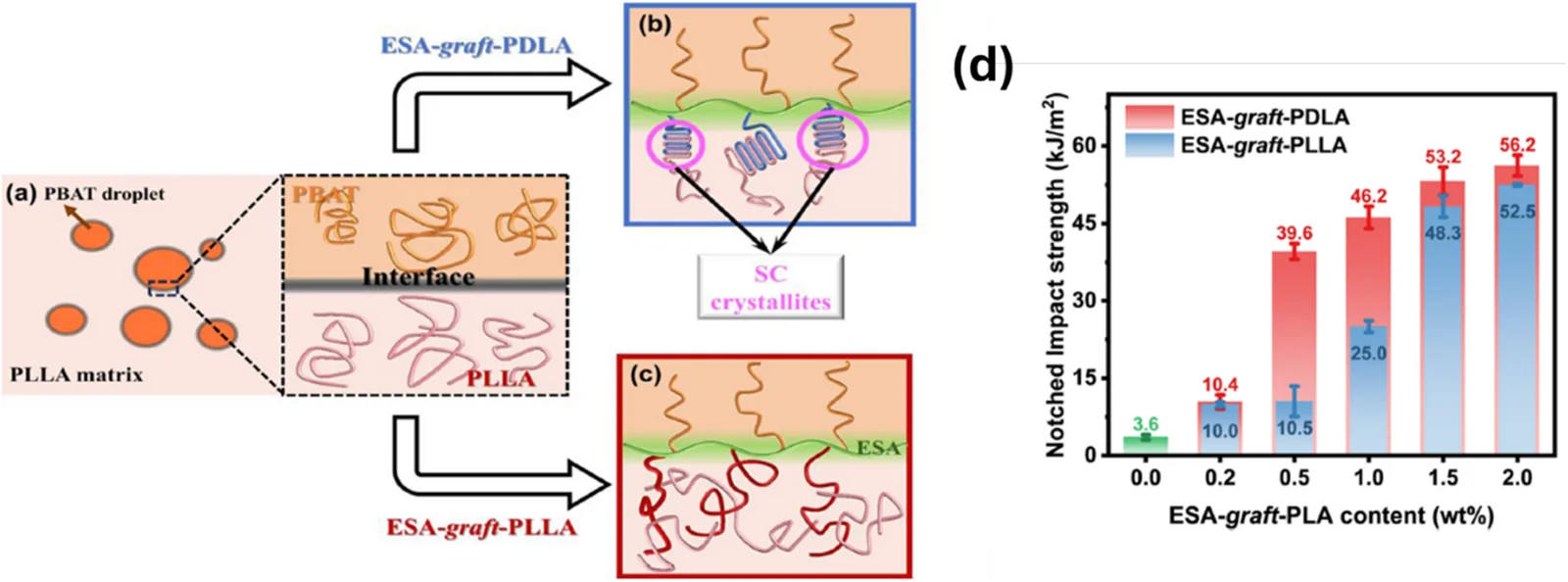
Schematic representation of the interfacial evolution in PLLA/PBAT blends induced by ESA-*graft*-PLA modifiers: (a) limited interfacial chain diffusion; (b) formation of SC crystallites and PBAT grafting; (c) formation of PBAT-*graft*-PLLA copolymers; and (d) variation in notched Izod impact strength of PLLA/PBAT (70/30) blends with ESA-*graft*-PLA content. Reproduced with permission from ref. 74. Copyright 2025. Elsevier Science Ltd.

While, composition-driven phase evolution controls domain size and connectivity and can induce transitions from matrix–droplet to co-continuous structures, interfacial localization and interfacial crystallization provide complementary routes for morphology refinement. Interfacial localization primarily controls domain size and dispersion by positioning the compatibilizer at the phase boundary, thereby suppressing coalescence and improving stress transfer. In contrast, interfacial crystallization combines morphology refinement with the formation of a reinforcing crystalline structure at the interface, which can additionally increase crystallinity and thermal stability. Thus, interfacial localization is particularly useful when fine and stable phase morphology is the primary objective, whereas interfacial crystallization is advantageous when morphology control needs to be accompanied by improved stiffness and thermal resistance. Such approaches, however, require an optimum level of interfacial modification, as excessive accumulation or crystallite formation can saturate interfacial sites and reduce the mechanical reinforcement.

Morphological refinement alone does not necessarily translate into improved tensile properties when interfacial adhesion remains limited. Aguerri *et al.*^75^ investigated the incorporation of ε-poly-l-lysine (EPL) into PHB/PCL blends for active food packaging. SEM images revealed a heterogeneous, phase-separated morphology in the control blend, characterised by disordered structures and spherical domains associated with the low miscibility of PHB and PCL. The addition of EPL produced a slight increase in morphological homogeneity, with fewer and smaller spherical domains and larger areas of continuous phases, suggesting improved phase dispersion and possible enhancement of blend miscibility. However, tensile strength increased only from 16.2 ± 0.8 MPa in the control to 16.3 ± 0.6 and 17.5 ± 1.3 MPa with 3 and 5% EPL, respectively, while elongation at break changed marginally from 10.0 ± 0.2% to 10.3 ± 0.2 and 10.6 ± 0.2%. These differences were not statistically significant. This limited mechanical response suggests that the morphological changes were insufficient to overcome the underlying interfacial limitations of the immiscible blend. Although smaller domains can reduce stress concentrations and improve stress distribution, effective load transfer also requires adequate adhesion between the phases. The persistence of phase separation, together with the absence of clear evidence of substantially strengthened interfacial bonding, may therefore explain why the observed morphological refinement did not yield significant mechanical improvements.

### 3.3 Reactive interfacial bonding

Morphology refinement increases interfacial contact and can improve stress transfer, but the resulting interface must also possess sufficient adhesion to sustain applied loads. This can be done using reactive compatibilizers that strengthen the phase boundary through interfacial chemical interactions and covalent bonding.

Reactive compatibilization can improve both strength and ductility by enhancing interfacial adhesion, although its effectiveness depends on compatibilizer concentration. Chen *et al.*^77^ compatibilized PHB/polycaprolactone (PCL) blends with varying concentrations of poly(methyl/amino)silsesquioxane (PMASQ) microspheres. At an optimum loading of 3 wt% PMASQ, tensile strength increased from 16.11 ± 1.09 to 19.81 ± 0.17 MPa, while elongation at break increased from 54.77 ± 8.91% to 337.82 ± 22.25%. The improvement was attributed to enhanced interfacial interactions and covalent bonding between PMASQ and the polymer chains. SEM analysis also revealed a more homogeneous morphology with reduced dispersed-phase size, while crystallinity decreased to 33.01%. However, increasing PMASQ to 5 wt% diminished the mechanical benefits due to poorer dispersion and increased phase aggregation of PMASO. The compatibilizer concentration is crucial to balance interfacial adhesion, morphology and mechanical performance.

The addition of a ductile phase does not necessarily improve the toughness in immiscible blends. Laoutid *et al.*^78^ addressed this limitation in PHB/PCL blends through reactive melt processing with dicumyl peroxide (DCP). For the 60/40 PHB/PCL blend, adding 5 wt% DCP increased elongation at break from 2.7% to 5.1% and impact resistance from 1.7 to 7.2 kJ m^−2^. The authors attributed these improvements to reactive grafting and crosslinking, accompanied by refinement of the blend morphology. Subsequent addition of 5 wt% polyethylene glycol (PEG) reduced the Young's modulus from 680 to 640 MPa and further increased impact resistance to 15.4 kJ m^−2^. The limited improvement obtained with PEG alone suggests that plasticisation was more effective when combined with reactive modification. Epoxidized cardanol-grafted PLA (PLA-*g*-ECA) have been used to compatibilize PLA/PBAT blend.^76^ PLA-*g*-ECA preferentially reacted with PBAT through epoxy ring-opening reactions, thus forming grafted copolymers at the interface (Fig. 4). This improved compatibility between the phases (reflected by the reduced difference in glass transition temperatures). As a result, the PBAT domain size decreased from ∼0.55 µm to ∼0.43 µm at the optimal 10 wt% PLA-*g*-ECA. This led to a finer and more uniform morphology that promoted better stress transfer between phases. Consequently, both ductility and toughness improved significantly, with % EAB increasing from 93% to 251% and IS increasing from 9.6 to 12.5 kJ m^−2^ (Table 1, S. no. 11). These improvements were mainly attributed to the formation of stronger interfacial bonding through epoxy-mediated reactions between PLA-*g*-ECA and PBAT, which improved adhesion between the two phases and reduced interfacial debonding under applied stress. The grafted copolymer formed at the interface also promoted greater chain entanglement across the phase boundary, facilitating more effective stress transfer between PLA and PBAT. Consequently, the finer and more strongly bonded morphology allowed the blend to undergo greater plastic deformation before failure, while interfacial deformation and chain mobility contributed to greater energy dissipation during loading.

**Fig. 4:**
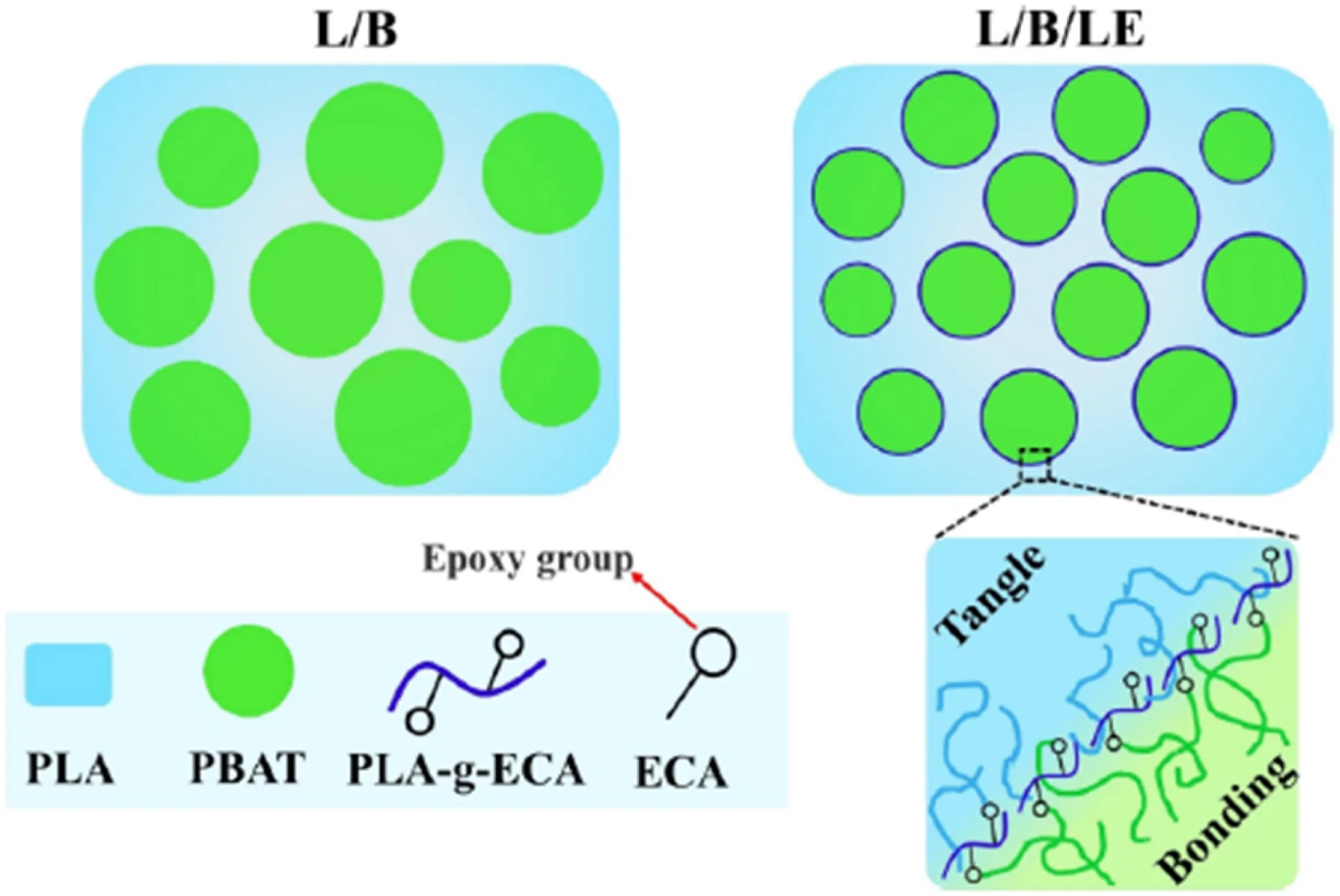
Illustration of interfacial morphology development in PLA/PBAT blends induced by PLA-*g*-ECA compatibilization. Reproduced with permission from ref. 76. Copyright 2024, American Chemical Society.

The effectiveness of compatibilisation also depends on copolymer architecture. Jeepery *et al.*^79^ examined an atactic PHB-*b*-PCL diblock copolymer as a compatibiliser for immiscible PHBH/PCL blends. In the 80/20 PHBH/PCL blend, the copolymer reduced the size of dispersed PCL domains and improved mechanical performance, with elongation at break increasing as the copolymer content increased. However, its addition produced only minor changes in morphology and mechanical properties in the 60/40 blend, indicating that the same compatibilisation strategy was less effective at this composition. When used directly as a blend component, the block copolymer also substantially increased the flexibility of PHBH. The 80/20 PHBH/BCP blend reached an elongation at break of 480%, compared with 7.7% for neat PHBH, while its maximum stress decreased from 27.4 to 17.8 MPa. Thus, there is a trade-off between flexibility and stress-bearing capacity.

The effectiveness of reactive compatibilization is also determined by the molecular characteristics (molecular weight and grafting density) of the compatibilizer can also influence its interfacial localization and anchoring. Miao *et al.*^80^ demonstrated how the molecular weight and grafting ratio of maleic anhydride-grafted PBAT (MA-*g*-PBAT) affect the compatibility and mechanical properties of PLA/PBAT blends. MA-*g*-PBAT improved interfacial adhesion and refined the blend morphology. Reactive grafting promoted the formation of interfacial copolymers, which reduced interfacial tension and helped MA-*g*-PBAT localize at the phase boundary. This localization can be justified thermodynamically as the compatibilizers partition toward the interface, they lower the relevant interfacial free energy with respect to its distribution within the bulk phases. (Δ*G*_localization_ = Δ*G*_PLA_ + Δ*G*_PBAT_ − Δ*G*_PLA–PBAT_) associated with unfavourable PLA/PBAT contacts,^81^ Since the PBAT backbone preferentially interacts with the PBAT phase while the grafted maleic anhydride groups provide favourable interactions with PLA. Consequently, the compatibilizer localizes at the interface, where it lowers the effective interfacial tension and suppresses phase coalescence. This stabilized the morphology, producing smaller (∼2.5 µm at optimal loading) and more uniformly dispersed PBAT domains. This improved morphology significantly enhanced toughness. At 3 wt% compatibilizer loading, % EAB increased from 7.1% to more than 233%, while IS increased nearly fourfold (∼37.1–43.0 kJ m^−2^). At the same time, the tensile modulus (∼1.7 GPa) and yield strength (∼47 MPa) remained almost unchanged (Table 1, S. no. 12). This indicates that toughness improved without sacrificing stiffness and strength. The compatibilization effect was stronger for MA-*g*-PBAT with higher molecular weight because it provided better interfacial anchoring and lower interfacial tension. However, excessive MA-*g*-PBAT content (8 wt%) caused phase separation and interfacial defects, which reduced mechanical performance.

In multiphase systems containing more than two polymers, the compatibilizer must be selectively positioned at the appropriate phase boundary. Zhu *et al.*^82^ investigated this concept in core–shell ternary PLA/polyglycolide (PGA)/PBS blends by engineering the location of the bio-based compatibilizer ESO3-ECD. ESO3-ECD preferentially localized at the PBS/PGA interface and formed covalent bonds through epoxy–carboxyl interactions, which improved interfacial adhesion and stabilized the core–shell morphology. As a result, stress was distributed more evenly across the blend and stress concentration at the phase boundaries was reduced. The optimized blends showed significant improvements in both strength and ductility, with TS increasing from 36.1 MPa to 46.9 MPa and % EAB increasing from 4.0% to 20.0% (Table 1, S. no. 16). In contrast, Joncryl ADR produced only limited improvements because it remained distributed within the PLA phase instead of localizing at the PBS/PGA interface. This shows that effective compatibilizer placement at the interface is important for improving interfacial reinforcement and stress transfer in multiphase blends.

These studies demonstrate that mechanical performance of the blends not only depends on the presence of reactive groups but also on compatibilizer molecular architecture and its selective positioning at the phase boundary. However, the existing studies largely establish localization after blending through morphology and interfacial characterization. For example, MA-*g*-PBAT was found to localize at the PLA/PBAT interface, while ESO3-ECD preferentially localized at the PBS/PGA interface. In contrast, Joncryl ADR remained predominantly within the PLA phase in the ternary system. But, why does a particular compatibilizer localize at a particular interface, and can this localization be predicted before processing?

A more predictive understanding of reactive interfacial bonding requires coupling interfacial thermodynamics with reaction kinetics to establish how compatibilizer structure and processing conditions govern its localization, interfacial bonding, and subsequent phase morphology. Such an approach could enable targeted design of compatibilizers for specific interfaces, particularly in multiphase biodegradable blends where multiple competing interfaces exist.

### 3.4 Chain extension and crosslinking

Multifunctional chain extenders and crosslinking agents can modify the polymer structure during blending, in contrast to acting solely at the interface. Such approaches provide a control over blend performance by simultaneously influencing molecular weight, branching, network formation, and the deformation behaviour of the material. Yan *et al.*^34^ investigated the effect of the epoxy-based chain extender poly (styrene-acrylic-*co*-methacrylate glycidyl) (ADR) and PBS content (0–30%) on the properties of the composites of chitosan (CS), PLA, and PBS. The addition of PBS significantly influenced the tensile, bending, IS, and % EAB of the blends. With increasing PBS content, the tensile strength decreased from 71.40 MPa (0% PBS) to 48.25 MPa (30% PBS), and the bending strength declined from 107.15 MPa to 74.30 MPa. In contrast, the % EAB initially increased, reaching a maximum of 159.02% at 20% PBS, before decreasing at higher loadings (Table 1, S. no. 8). Similarly, the IS showed an initial improvement followed by a reduction at elevated PBS contents. Increasing PBS content increased chain mobility and promoted cavitation, shear-band formation, and energy dissipation. This improved ductility and impact resistance. However, the higher PBS content also reduced tensile and bending strength. ADR helped compensate for this loss in mechanical strength. Its epoxy groups reacted with the amino, carboxyl, and hydroxyl groups of CS, PLA, and PBS, respectively. These reactions formed a crosslinked network and strengthened the interactions between the constituent phases. The resulting network improved stress transfer and helped maintain the load-bearing structure of the blend. Thus, PBS improved toughness, while ADR helped retain strength and stiffness. This combination reduced the stiffness–toughness trade-off and resulted in the best overall mechanical response at 15–20% PBS.

Using compatibilizing agents to simultaneously modify the polymer network and phase interface is another interesting approach with practical results. Dicumyl peroxide (DCP) and methyl methacrylate-glycidyl methacrylate copolymer (P(MMA-*co*-GMA)) have been reported to modify the morphology and mechanical properties of PLA/PBAT blends using this principle.^83^ In unmodified systems, the presence of large PBAT “sea-island” domains within the PLA matrix indicated poor miscibility, leading to reduced tensile strength despite improved ductility. As shown in the schematics in Fig. 5(a), poor interfacial compatibility promoted debonding and crazing within the PLA matrix under tensile loading, resulting in premature failure. The addition of the reactive agents DCP and P(MMA-*co*-GMA) significantly improved interfacial interactions in the blends. DCP promoted partial crosslinking through free-radical reactions, which reduced the size of PBAT domains and improved interfacial adhesion. Meanwhile, P(MMA-*co*-GMA) reacted with PLA and PBAT through epoxy ring-opening reactions, forming branched or partially crosslinked structures that further refined the morphology into smaller and more elongated domains with larger interfacial area. As shown in Fig. 5(b), improved compatibility enables more effective stress transfer across phases, suppressing interfacial debonding and promoting plastic deformation. AFM nanomechanical mapping confirmed the formation of interfacial transition zones and an increase in the local EM of the PBAT phase, indicating improved chain entanglement and stress transfer. Consequently, these morphological refinements translated into enhanced mechanical properties, with compatibilized blends showing a slight recovery in TS and a significant increase in % EAB and toughness, particularly at higher compatibilizer loadings (*e.g.*, 10 phr) (Table 1, S. no. 10).

**Fig. 5:**
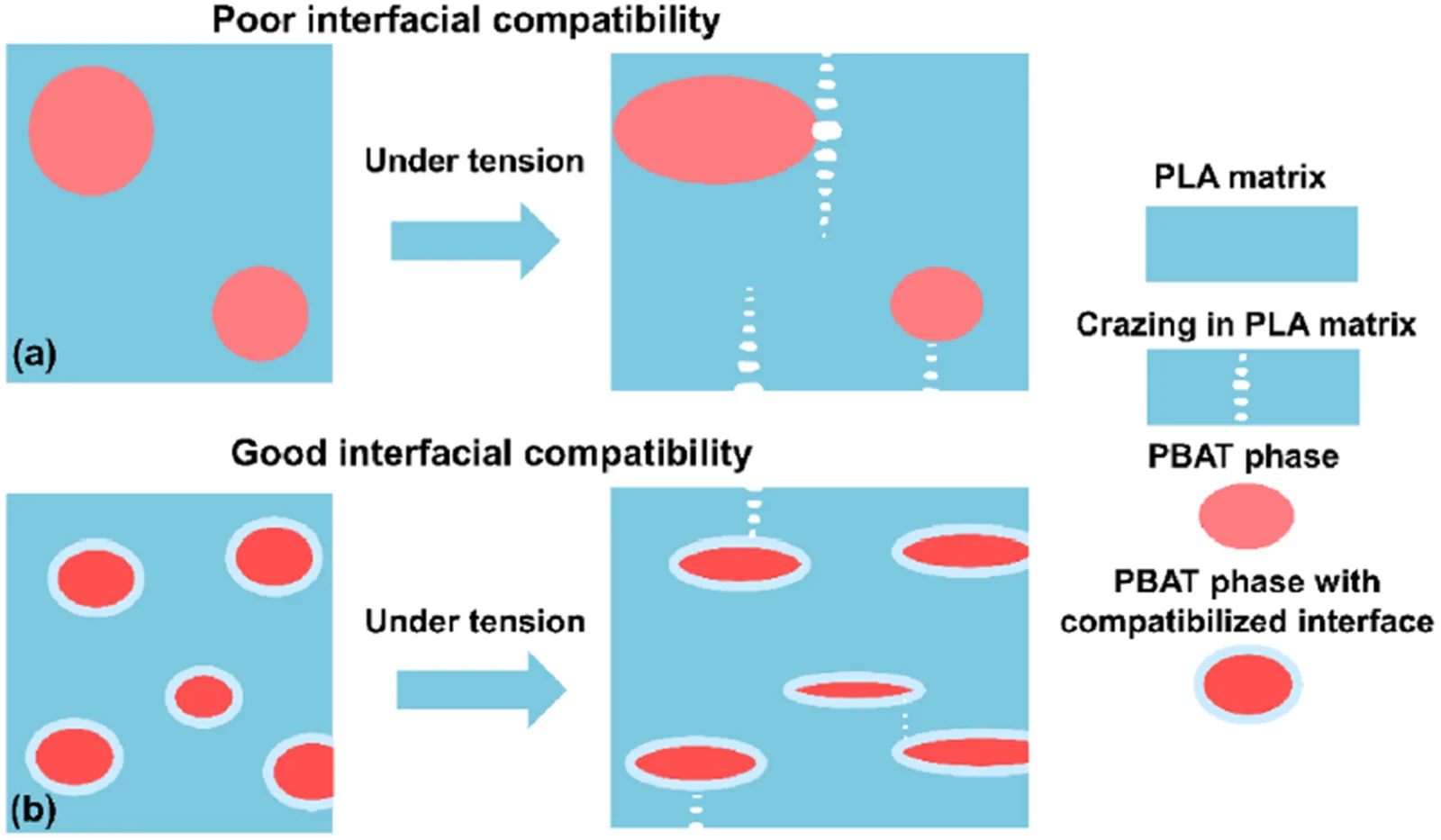
Schematic illustration of the effect of interfacial compatibility on the crazing in PLA-based blends: (a) poor interfacial adhesion; (b) improved interfacial adhesion. Reproduced with permission from ref. 83. Copyright 2024. Elsevier Science Ltd.

The studies discussed above show that reactive chain extension, branching and crosslinking provide additional level of control beyond conventional morphology and interfacial modification. Chain extension primarily increases molecular weight and the studies discussed above show that increasing chain connectivity; branching can enhance melt strength and entanglement; cross linking introduces network constraint that can improve dimensional stability and resistance to deformation. Increasing connectivity from these three distinct molecular modification can improve strength while simultaneously restricting chain mobility reducing % EAB. The key challenge is therefore not simply to increase chain connectivity or crosslink density, but to control the extent of modification so that strength, toughness, and deformability are balanced.

### 3.5 Filler reinforcements

Organic and inorganic fillers can also significantly influence the microstructure and mechanical properties of polymer blends by imparting their inherent characteristics. Fillers such clay, fibres, and secondary biopolymer components can strongly affect morphological evolution. These additives introduce a range of interactions that may either stabilise or destabilise the blend morphology, thereby impacting its thermal and mechanical properties.

Polymer–filler wetting theory provides a thermodynamic framework for understanding how filler surface chemistry can influence its preferential localization within an immiscible polymer blend (eqn (5)). However, experimental studies do not always directly quantify filler localization or the corresponding interfacial tensions. Mtibe *et al.*^84^ demonstrated the effect of lignin (5 to 15 wt%) and ZnO nanoparticle (1.5 wt%) on the mechanical properties of PBS/PBAT blends at the ratio of 70 : 30. FTIR analysis indicated non-covalent interactions between lignin and the polymer blend, suggesting interactions between the filler and the polymer matrix. Increasing lignin content reduced TS and % EAB but increased tensile modulus. At 15 wt% lignin, tensile strength and elongation at break decreased by ∼18% and ∼167%, respectively. The tensile modulus increased by ∼31% to 347.44 MPa (Table 1, S. no. 14). The reduction in strength and ductility was due to the low aspect ratio and aromatic structure of the lignin, while the increase in stiffness was associated with the incorporation of rigid filler particles. The study demonstrates how polymer–filler interactions and filler rigidity can modify the mechanical response of an immiscible polymer blend, although the preferential localization of lignin or ZnO within the PBS/PBAT phases was not directly evaluated.

Donitz *et al.*^85^ demonstrated how the hemp fibre length influences the mechanical properties of PBS. They considered short (2 mm) and long (10 mm) hemp fibres at the same weight percentage with respect to PBS (5 wt%). The incorporation of hemp fibres, including both short fibre (SF) and long fibre (LF), led to a significant increase in the stiffness of PBS, with the EM rising from 510 MPa (PBS) to 820 MPa (SF-PBS) and 840 MPa (LF-PBS). This enhancement is attributed to the rigid nature of the fibres and improved stress transfer, which is considerably more effective in the case of long fibres. However, the TS decreases after addition of hemp fibre, dropping from 35.5 MPa (PBS) to 27.0 MPa (SF-PBS) and further to 24.0 MPa (LF-PBS), indicating increased stress concentration and interfacial limitations. Both SF and LF increased the stiffness of PBS but reduced its strength. LF showed a slightly higher EM but caused a greater reduction in TS than SF. Thus, filler geometry, along with filler loading and rigidity, can influence stress transfer and the mechanical response of biodegradable polymer composites.

The incorporation of fillers into biopolymer blends should consider filler loading, size, geometry, dispersion, surface chemistry, and polymer–filler interactions. However, most studies primarily evaluate the resulting mechanical properties and filler–polymer interactions, while the thermodynamic basis of filler localization and its direct relationship with blend morphology are less frequently quantified. Future studies should therefore combine interfacial thermodynamics with experimental characterization of filler localization and processing effects to establish predictive relationships between filler characteristics, spatial distribution, morphology, and mechanical response.

### 3.6 Processing-induced morphology

Processing parameters such as temperature, shear rate, mixing time, residence time, and melt viscosity influence phase breakup, coalescence, interfacial diffusion, and reactive interactions. These factors can therefore determine the size, distribution, and connectivity of the dispersed phases, as well as the extent of chain extension or degradation during reactive blending. Consequently, similar blend compositions and compatibilizer contents can produce different morphologies and mechanical responses under different processing conditions. Understanding how processing conditions control the evolution of morphology is therefore essential for establishing reliable structure–property relationships in biodegradable polymer blends.

Altinbay *et al.*^86^ reported on the Joncryl epoxy-based chain extender and observed how it affects the reactivity and performance of PLA/PBAT blends processed at various temperatures, various aged PBAT. The mechanical properties of the PLA/PBAT blends were considerably influenced by processing temperature, PBAT aging, and the addition of the Joncryl chain extender through their combined effects on molecular structure, rheology, and morphology. Joncryl enhanced compatibilization by reacting with both PLA and PBAT, increasing molecular weight and improving interfacial adhesion, which led to higher % EAB, toughness, and IS, particularly at lower processing temperatures. Increasing processing temperature further promoted Joncryl reactivity and transesterification, resulting in improved rheological properties and a finer phase morphology (Table 1, S. no. 9). However, excessively high processing temperatures promoted PLA degradation, leading to a slight decrease in TS. Thus, the processing temperature must be balanced to promote Joncryl reactions and morphology refinement while avoiding thermally induced polymer degradation.

The morphology of biodegradable polymer blends can be altered by the shear history imposed during melt processing, even when the blend composition and residence time are kept constant. Gigante *et al.*^87^ investigated PLA/PBSA blends processed by twin-screw extrusion at screw speeds of 300, 350, and 400 rpm. Increasing the screw speed reduced the PBSA droplet size and increased the interfacial area between the PLA and PBSA phases. At 400 rpm, the dispersed-phase radius predicted by the extrusion model was approximately 0.16 µm, while SEM analysis confirmed the formation of smaller PBSA droplets at higher screw speeds. The finer morphology also resulted in higher melt viscosity at a given shear rate, with the 400 rpm samples showing the highest viscosity. For the PLA75/PBSA25 composition, the change in screw speed produced the clearest variation in elongation at break, linking the processing-induced reduction in dispersed-phase size with the mechanical response. Thus, increasing shear during extrusion provided a means of refining the dispersed phase and modifying the rheological and mechanical behaviour of PLA/PBSA blends.

The effect of processing conditions on phase dispersion is also evident in PBAT/TPS blends, where the morphology depended on the shear and mixing conditions during compounding. Genovesi *et al.*^88^ varied the screw speed during twin-screw compounding of PBAT/TPS blends before cast-film extrusion and evaluated the resulting morphology, mechanical behaviour, and film processability. The study showed that the quality of TPS dispersion was strongly dependent on screw speed, with the resulting differences in phase dispersion affecting the quality and processability of the extruded films. Thus, the mechanical and processing performance of PBAT/TPS blends can be modified through control of the compounding conditions rather than through changes in blend composition alone.

Processing conditions can also control the structure of multilayer biodegradable films and thereby affect their barrier and mechanical performance. Palmieri *et al.*^89^ produced PBS/PBSA/PHB multilayer blown films containing 0, 2, and 5 wt% Cloisite 30B in the PHB functional layer, while varying the functional-layer screw speed from 30 to 60 rpm. Increasing the screw speed increased the functional-layer thickness from 0.059 to 0.072 mm for neat PHB and from 0.060 to 0.075 mm for PHB containing 2 wt% nanoclay (PHB2). The PHB2 film processed at 30 rpm showed an elongation at break of 29%, while the film processed at 60 rpm exhibited the lowest oxygen permeability, at 2.92 cm^3^ mm m^−2^ day^−1^ bar^−1^. The addition of 2 and 5 wt% Cloisite 30B also reduced the crystallinity of the PHB functional layer from 46.1% for melt-processed PHB to 42.3% and 32.3%, respectively. At 5 wt% nanoclay, poor bubble stability prevented collection of films at 50 and 60 rpm, demonstrating the combined effect of formulation and processing conditions on film formation. Thus, extrusion conditions can regulate functional-layer thickness and, together with nanofiller incorporation, alter the mechanical performance of PBS/PBSA/PHB multilayer films.

These studies show that processing conditions affect biodegradable polymer performance through several mechanisms. These include reactive modification, shear-induced phase dispersion, layer formation, and changes in crystallinity. Higher shear can refine dispersed domains and change the interfacial area. Processing temperature must also be controlled to promote interfacial reactions while avoiding thermal degradation. In multilayer systems, processing conditions affect layer thickness and film stability, which in turn influence barrier and mechanical properties. Therefore, processing effects depend on molecular reactions, rheology, crystallization, and blend composition. An optimum processing window is needed to obtain the desired morphology and functional properties.

## 4 Barrier properties

Barrier properties signify the ability of a material to resist gases', vapours', or liquids' diffusion. These are relevant for applications, such as food packaging which requires controlled oxygen and moisture transfer to enhance product shelf life and stability. Barrier properties include parameters such as oxygen permeability (OP) and water vapor transmission rate (WVTR).

Oxygen and water vapor transport through polymer films generally follows the solution–diffusion mechanism,^94–96^ involving sorption of the permeant into the polymer, diffusion through the polymer, and subsequent desorption. However, the two permeants differ substantially in their interactions with the polymer matrix. Oxygen is relatively nonpolar and has limited specific interactions with most polymer chains. Its permeability is therefore primarily associated with its ability to dissolve in and diffuse through the polymer. In contrast, water vapor is highly polar and can form specific interactions, particularly hydrogen bonds, with polar or hydrophilic functional groups in the polymer. Therefore, oxygen barrier performance is primarily associated with the diffusional transport of a weakly interacting, nonpolar molecule, whereas water-vapor barrier performance involves both diffusion and the thermodynamic affinity between water and the polymer. This difference makes WVTR particularly sensitive to the chemical functionality and hydrophilicity of the polymer, whereas oxygen permeability is more directly associated with the polymer's ability to accommodate and transport oxygen molecules.

These differences in water vapor/oxygen transport in polymeric materials provide a basis for understanding how changes in blend composition polymer composition, phase structure, interfacial interactions, and the incorporation of additives and fillers influence oxygen and water-vapor barrier performance. The following sections therefore examine how these factors influence these transport processes in biodegradable polymer blends.

### 4.1 Blend composition, morphology, and molecular packing

Initial studies on biodegradable polyester blends showed that barrier performance is affected by phase morphology, crystallinity, and polymer chain packing, as these factors control the diffusion pathways for gases and moisture. Coltelli *et al.*^97^ demonstrated this by investigating the barrier performance of PLA/PBSA biodegradable films by varying the PLA and PBSA composition. Among the compositions studied, the 70% PBSA blend showed the lowest weight loss and the best moisture resistance. Because the more apolar nature of PBSA reduced the diffusion of water molecules through the film. In contrast, the 60% PBSA blend exhibited the poorest barrier performance due to its coarse morphology, large dispersed domains, and weak interfacial adhesion, which increased water transport. Co-continuous structures, such as the 40% PBSA blend, showed intermediate performance. The homogeneous PBSA-rich blends restricted diffusion pathways more effectively. Additionally, moisture loss exhibited a near-linear trend over time, suggesting the rapid attainment of steady-state diffusion with short lag times, comparable to conventional materials such as low density polyethylene (LDPE). These results demonstrate that moisture barrier performance is governed by the combined effects of polymer polarity and phase morphology and not blend composition alone. Further, homogeneous and finely dispersed morphologies can restrict moisture diffusion, whereas coarse heterogeneous structures with large domains and weak interfacial adhesion can facilitate water transport. A trade-off therefore exists between mechanical reinforcement and barrier performance. Co-continuous and interconnected morphologies can enhance stiffness by providing continuous load-bearing pathways and facilitating stress transfer between phases, but the same interconnected structure may also create continuous pathways for moisture or gas transport. In contrast, homogeneous and finely dispersed morphologies can restrict permeant diffusion more effectively, although they may provide less efficient load transfer than an interconnected network. Therefore, blends intended for applications requiring both stiffness and barrier performance should not be designed solely to maximize either phase continuity or dispersion. Instead, the phase morphology, domain size, interfacial adhesion, phase connectivity, and continuity of the diffusion pathways must be considered together to identify a morphology that provides an appropriate balance between mechanical load transfer and resistance to gas/moisture transport.

The molecular structure and packing of the constituent polymers can also influence barrier performance. Threepopnatkul *et al.*^28^ demonstrated this by studying the effect of essential oils encapsulated in β-cyclodextrin (βCD-ES) on the water vapour and oxygen barrier properties of PBAT/PBS blends. The barrier properties of PBAT/PBS films varied with both polymer composition and the presence of βCD-ES. Increasing PBS content improved barrier performance (Fig. 6(a)), due to the enhanced crystallinity. The formation of a more crystalline structure increased the degree of molecular ordering and reduced the volume of amorphous regions available for permeant transport. The resulting reduction in free volume limited the mobility of oxygen and water molecules through the polymer matrix, thereby decreasing both oxygen permeability (OP) and water vapor transmission rate (WVTR). The incorporation of βCD-ES had the opposite effect, leading to increased values of WVTR and OP (Fig. 6(b)). This was attributed to the plasticizing action of the essential oil and the presence of β-cyclodextrin, which disrupted polymer packing and created additional free volume, which increased gas and moisture diffusion. Although encapsulation within βCD helped control the evaporation of the essential oil and maintain its presence within the matrix, it did not mitigate the increase in permeability caused by reduced intermolecular interactions. Thus, while PBS improved barrier performance, the introduction of βCD-ES compromised it by increasing chain mobility and diffusion pathways. These results demonstrate that improvements in barrier performance arising from crystallinity can be offset by additive that disrupt molecular packing or increase chain mobility.

**Fig. 6:**
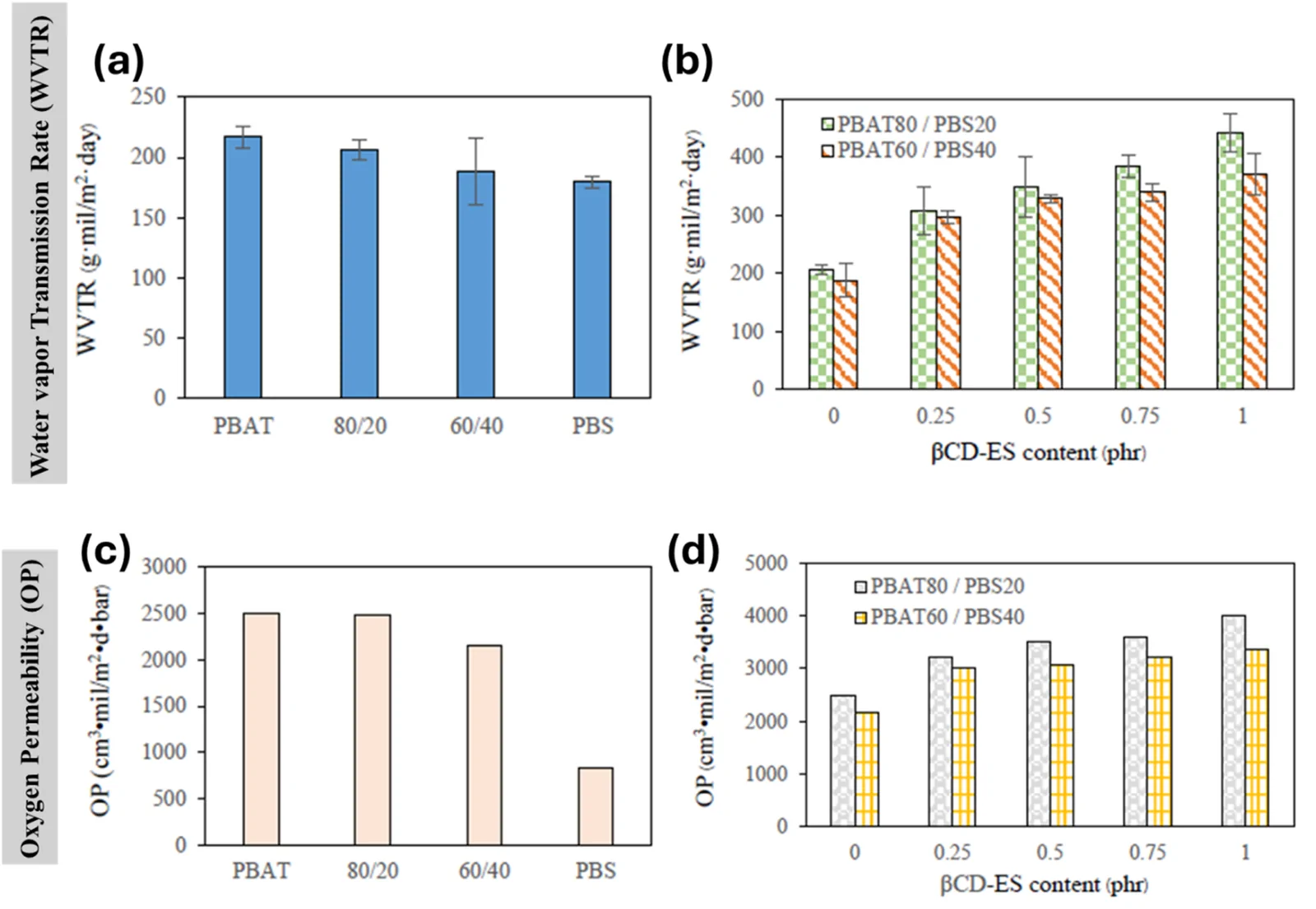
Water vapor transmission rate (WVTR) of PBAT/PBS films: (a) without βCD-ES and (b) with βCD-ES; oxygen permeability (OP) of PBAT/PBS films: (c) without βCD-ES and (d) with βCD-ES. Reproduced under the terms of the Creative Commons Attribution License from ref. 28. Copyright 2022. IOP Publisher.

### 4.2 Interfacial compatibility and reactive modification

Interfacial compatibilization is an important strategy for significantly improving barrier performance by reducing interfacial defects and promoting tortuous diffusion pathways. For example, Adrar *et al.*^93^ reported the effect of epoxy-functionalized ethylene-methyl acrylate-glycidyl methacrylate (ax89) compatibilizer and organo-montmorillonite D43B clay on the oxygen barrier properties of PBS/PLA-based nanocomposite blown films. Blending order dictated the localization of the D43B nano-clay and the extent of compatibilization, which in turn controlled morphology evolution from nodular to co-continuous structures. SEM and TEM analyses showed that the blending sequence influenced phase morphology, interfacial adhesion, and nanofiller distribution. The addition of ax89 promoted co-continuous morphologies with fewer cavities and improved interfacial adhesion, while D43B preferentially localized within the PLA phase. XRD analysis further confirmed the formation of intercalated clay structures, which increased the tortuous diffusion pathways for gas molecules. In contrast, unsuitable blending sequences resulted in phase separation and non-uniform filler dispersion, limiting the improvement in barrier performance. As a result, the immiscible PBS/PLA blend showed higher OP than the neat polymers. The addition of ax89 reduced OP by ∼38% due to improved compatibility, while D43B further enhanced the barrier performance. The combined use of ax89 and D43B produced the greatest reduction in OP (∼53%).

Interestingly, the effect of co-continuous morphology on the barrier performance of the blend differs between moisture and oxygen transport. Coltelli *et al.*^97^ reported intermediate moisture barrier performance for the co-continuous PLA/PBSA structure, whereas Adrar *et al.*^93^ observed improved oxygen barrier performance in a co-continuous PBS/PLA morphology. This difference reflects the distinct transport mechanisms of water vapor and oxygen. Water transport is governed by the affinity of water for the polymers and its ability to sorb into the matrix, whereas oxygen transport is primarily governed by its dissolution and diffusion through the polymer. Therefore, the effect of a given phase morphology cannot be generalized across different permeants of polymer systems, as the same structural feature may influence their transport differently.

Further studies explored more advanced interface engineering approaches in ternary systems to improve the interfacial compatibility and barrier performance of the blend. Zhu *et al.*^82^ developed core–shell PLA/PGA/PBS ternary blends and reported significant improvements in oxygen barrier properties. The addition of the ESO3-ECD chain extender reduced OP from ∼0.17 Barrer in the unmodified blends to ∼0.14 Barrer, outperforming ADR-modified systems (∼0.15 Barrer). The reduction in OP was achieved through the preferential localization of ESO3-ECD at the PBS/PGA interface, where its epoxy groups reacted with carboxyl groups to strengthen interfacial adhesion and stabilize the core–shell morphology. The resulting phase structure increased the tortuosity of the oxygen diffusion pathway, restricting oxygen transport (Fig. 7(a)). Additionally, the optimized blends exhibited a favourable permeability ratio (≈0.33), enabling the formation of a low-O_2_/high-CO_2_ internal environment beneficial for food preservation (Fig. 7(b) and (c)). Interfacial compatibilization had only a small effect on the water vapour permeability. Water-vapor transport was governed by the affinity of water for the hygroscopic PGA phase. Thus, controlling phase morphology and interfacial adhesion may be particularly effective for reducing oxygen transport. Whereas, improving water-vapor barrier performance requires additional control over the hydrophilicity and water–polymer interactions of the constituent phases.

**Fig. 7:**
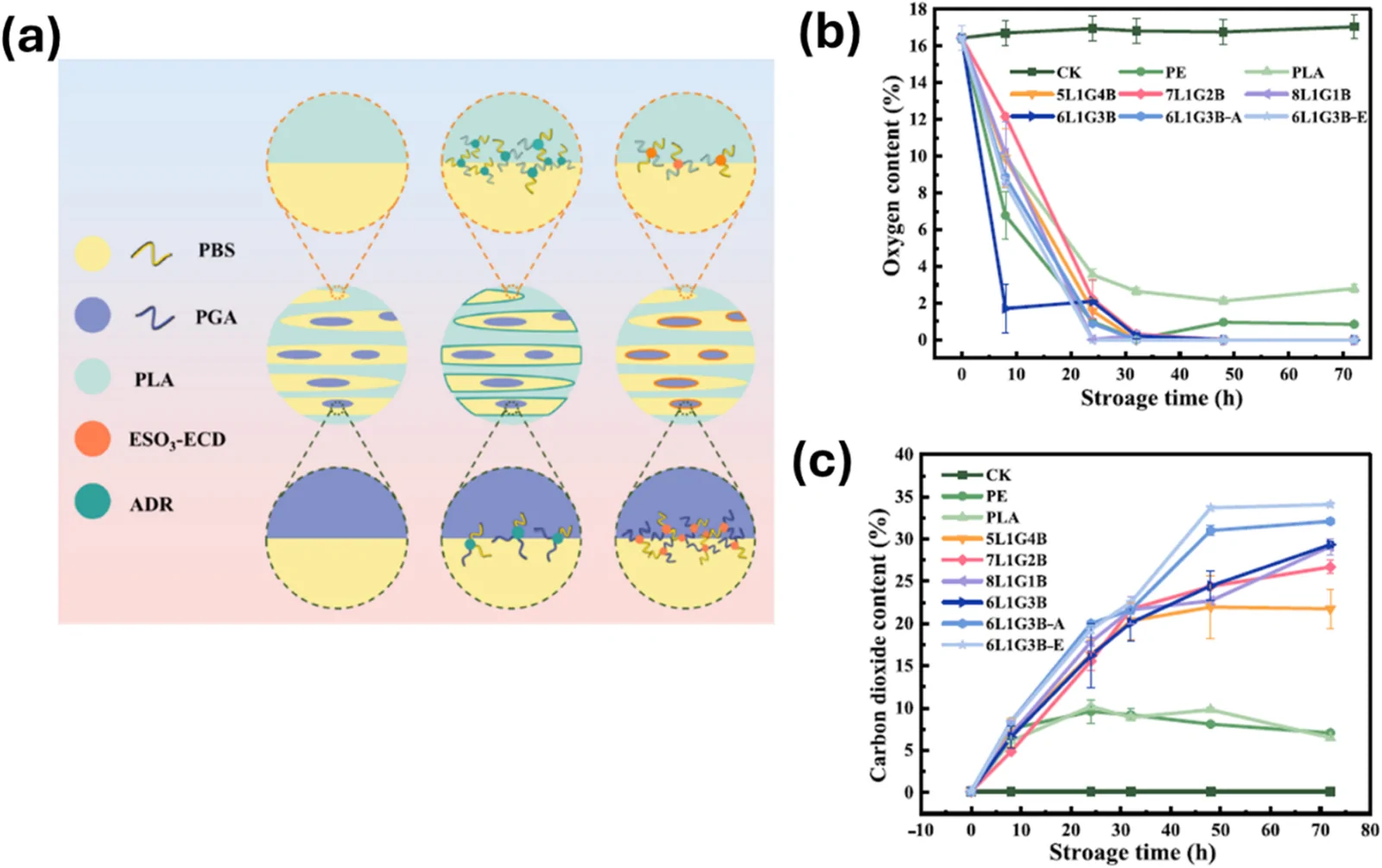
(a) Schematic illustration of compatibilization mechanisms of ADR at the PLA/PBS interface and ESO3-ECD at the PBS/PGA interface; (b) evolution of O_2_ concentration; and (c) evolution of CO_2_ concentration during broccoli storage. Reproduced with permission from ref. 82. Copyright 2025. Elsevier Science Ltd.

### 4.3 Filler-induced tortuous diffusion pathways

More recent research has also focused on adding nanofillers to biopolymer to improve barrier performance. Delorme *et al.*^98^ modified PBSA films with SORBACID® 911 layered double hydroxide (LDH) filler and observed the changes in its gas and water vapour barrier properties. The incorporation of LDH introduced impermeable platelet-like regions within the PBSA matrix, forcing oxygen molecules to follow longer and more tortuous diffusion pathways rather than passing directly through the polymer. From a thermodynamic perspective, possibly the low affinity of oxygen for the inorganic LDH phase restricts its partitioning into these regions, while favourable PBSA–LDH interactions can alter local polymer packing and reduce the free volume available for molecular transport. These effects increase the resistance to oxygen diffusion through the composite. Consequently, oxygen transmission rate (OTR) gradually decreased with increasing LDH content. OTR reduced to ∼9% at 2 wt% and ∼16% at 5 wt% of LDH. WVTR also decreased significantly, with reductions of ∼55% at 2 wt% and ∼44–54% at higher loadings of LDH. Well-dispersed LDH provided a larger effective barrier and more uniformly distributed tortuous pathways. Notably, poorer dispersion and aggregation limited the barrier improvement at 8 wt% LDH.

A complementary study by Apicella *et al.*^99^ investigated the effect of bio-based microfibrillated cellulose (MFC)as a on the barrier properties of PLA/PBSA blend (plasticized with cardanol). The oxygen barrier improved significantly after adding MFC. At 0.75 wt% MFC, OP decreased by 28% from 18.4 to 13.3 cm^3^ mm (m^2^ day bar)^−1^. Uniformly dispersed MFC (due to cardanol) led to the formation of a more tortuous diffusion pathway between the polymer phases. Also, improved interfacial adhesion between the PLA/PBSA phases further reduced the likelihood of interfacial defects that could provide preferential pathways for oxygen transport. Thus, the structural effect of MFC dominated the oxygen-transport behaviour at the optimum loading. In contrast, water-vapour transport was not reduced to the same extent and showed a slight increase in permeability. Unlike oxygen, water molecules have a strong affinity for the hydroxyl-rich cellulose surface. The hydrophilic nature of MFC therefore increases the sorption of water within the composite, which can partially offset the reduction in diffusivity produced by the more tortuous morphology. The overall water-vapour permeability can therefore be viewed as a balance between reduced diffusivity due to the tortuous MFC network and increased water sorption due to the hydrophilic cellulose phase. Thus, barrier performance depended on both the structural effects of MFC and its hydrophilic nature. Optimal dispersion at 0.75 wt% MFC was critical for improving barrier performance. At higher loadings, MFC agglomerated and reducing the barrier performance.

Beyond conventional nanofillers, functionalized polyhedral oligomeric silsesquioxane (POSS) nanoclusters have also been investigated for improving gas and moisture barrier properties in biodegradable PBSA nanocomposites. Chabane *et al.*^100^ investigated the barrier performance of PBSA-based nanocomposites reinforced with POSS and ionic liquid-modified POSS (IL-*g*-POSS) (Fig. 8(a)), demonstrating a significant enhancement in both oxygen and water vapor barrier properties due to improved nanofiller dispersion and interfacial interactions. The incorporation of POSS nanoclusters introduced tortuous diffusion pathways that hindered gas transport. However, the extent of improvement was strongly dependent on the dispersion state of the nanofillers. Unmodified POSS exhibited poor dispersion and formed micron-sized aggregates, resulting in moderate barrier improvements, with water permeability reduced by ∼27% and OP by ∼57% (Fig. 8(b)). In contrast, IL-*g*-POSS nanocomposites, characterized by well-dispersed nanoscale clusters (10–100 nm), showed markedly superior performance, achieving reductions of up to 87% in water vapor permeability and 95% in OP. This improvement was attributed to enhanced interactions between IL-*g*-POSS and the PBSA matrix, which improved filler dispersion and reduced interfacial defects. The IL-modified systems also showed higher crystallinity and hydrophobicity, which further restricted gas diffusion. These comparison demonstrate that surface functionalisation can improve barrier properties by modifying their distribution and interaction with the polymer matrix.

**Fig. 8:**
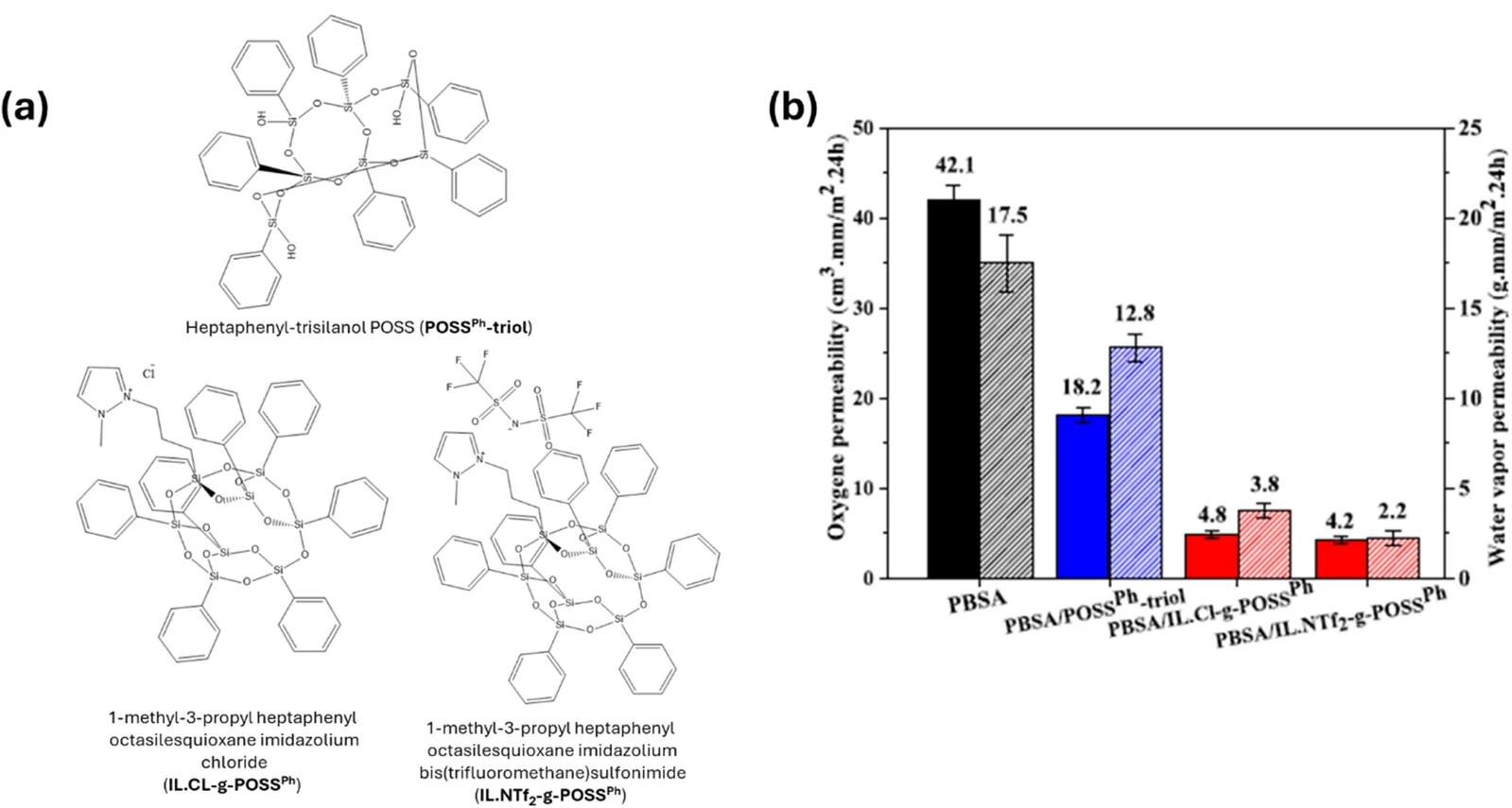
(a) Chemical structures of the compounds used to design nanostructured PBSA/POSS^Ph^ networks; (b) evolution of oxygen and water vapor permeability of neat PBSA and PBSA/POSS^Ph^-based nanocomposites. Reproduced with permission from ref. 100. Copyright 2024. American Chemical Society.

### 4.4 Processing-induced filler dispersion

The effectiveness of a filler in improving barrier properties depends not only on its intrinsic characteristics but also on its dispersion and spatial distribution within the polymer matrix. Processing determines whether the filler becomes uniformly dispersed, selectively localizes at a polymer–polymer interface, or forms aggregates. Well-dispersed fillers can generate continuous and effective tortuous pathways for gas and moisture transport, whereas agglomeration can introduce defects and less resistant transport regions.

Zhang *et al.*^73^ demonstrated the utility of hot pressing for improving the gas-barrier performance of PLA/PBS blends containing Janus nanosheets (JNs). The JNs preferentially localized at the PLA/PBS interface and formed continuous silica layers that restricted oxygen transport. In addition, hot pressing generated lamellar PBS domains and increased the diffusion path for gas molecules. Together, these structural changes reduced the oxygen permeability from 1330 Barrer to ∼80 Barrer at the optimal JN loading. However, at higher JN contents, excessive nanosheet accumulation at the interface formed multilayer structures that weakened the interface. As a result, the mechanical properties decreased, although the barrier performance continued to improve.

Similar improvements in barrier performance were reported in nanoclay-incorporated PBSA/PHBV blends, where processing method and filler dispersion played a crucial role in determining permeability reduction. Pesaranhajiabbas *et al.*^101^ modified the barrier properties of biodegradable PBSA/PHBV blends by incorporating nanoclay (Nanomer® I.30 T, surface modifier with *N*-octadecylamine) and using two different processing methods. The barrier properties of the PBSA/PHBV nanocomposites were influenced by both nanoclay addition and the processing method, as these factors controlled filler dispersion and phase morphology. The incorporation of nanoclay reduced oxygen and water vapor permeability by creating more tortuous pathways for gas diffusion. Even low nanoclay loadings (∼1 wt%) reduced oxygen permeability by 26–38%. However, the extent of barrier improvement strongly depended on how well the nanoclay was dispersed within the polymer matrix. Two-step processing (masterbatch approach) produced exfoliated and well-dispersed nanoclay layers, particularly localized at the PBSA/PHBV interface, which acted as physical barriers and improved phase adhesion. As a result, the lowest permeability values were achieved in two-step processed nanocomposites at ∼2 wt% nanoclay, with reductions of up to 36% (oxygen) and 35% (water vapor) compared to one-step processed systems. In contrast, one-step processing led to nanoclay agglomeration, limiting barrier improvements due to the formation of defects and relatively improved diffusion pathways. Furthermore, excessive nanoclay loading (≥3 wt%) resulted in increased permeability regardless of processing method due to agglomeration and void formation that promoted gas transport. Optimal nanoclay content and controlled two-step processing were critical to improving barrier properties.

Despite these advances, several experimental gaps remain. Barrier performance is often correlated with qualitative descriptions of morphology, while quantitative parameters such as domain-size distribution, phase connectivity, interfacial area, and filler localization are evaluated less consistently. A further challenge is that oxygen and water-vapour transport can respond differently to the same structural modification, as demonstrated by the competing effects of filler-induced tortuosity and hydrophilicity. Therefore, a predictive understanding of how molecular interactions and processing conditions determine interfacial structure, phase morphology, filler localization, and ultimately gas and moisture transport is still lacking. Establishing quantitative relationships between such structural parameters and the barrier properties would ultimately support the rational design of biodegradable packaging materials.

## 5 Rheological properties

Rheology explains how materials flow and deform when stress or strain is applied. It helps evaluate the processing and performance of polymeric materials.^102^ Some common rheological parameters are complex viscosity (η*), storage modulus (*G*′), and loss modulus (*G*″). These describe the viscous and elastic responses of the material. *G*′ is energy stored elastically during deformation, reflecting solid-like behaviour. *G*″ is the energy dissipated through viscous flow. The relative magnitude of *G*′ and *G*″ *vs.* angular frequency (*ω*) differentiates the solid- and liquid-like behaviour of polymer melts, respectively.^103^

The rheological behavior of biopolymers is controlled by their molecular properties, including molecular weight, chain flexibility, branching, and entanglement. Temperature, shear rate, and processing conditions also influence their rheological behaviour.^104^ The rheology of polymer blends and nanocomposites is further affected by phase morphology, interfacial interactions, and filler dispersion. These factors control chain mobility and relaxation.^105^ For example, compatibilization or stronger filler–polymer interactions can restrict chain motion and increase melt elasticity. In contrast, plasticization or phase separation increases chain mobility and reduces viscosity.

Understanding of the rheology of a material is crucial in processing it through techniques such as extrusion,^106^ injection molding,^107^ and fused filament fabrication (FFF).^108^ The melt flow behaviour of the material dictates the process stability and product quality. The material should have suitable viscosity and melt strength to maintain its shape during the processing. Rheological properties are also linked to phase morphology of the blend and the inter-polymer compatibility. Variations in viscosity and viscoelastic behaviour can depend on factors, such as phase dispersion, network formation, and interfacial adhesion. Therefore, rheology of the blend links the molecular structure, processing behaviour, and their final properties. The following sections discuss the effects of blend composition, interfacial interactions, reactive modification, and filler incorporation on rheological behaviour and processability of biodegradable polymer blends.

### 5.1 Blend composition, plasticization and phase morphology

Yan *et al.*^34^ explored the plasticizing effect of PBS content in the CS/PLA-ADR-PBS blends. Dynamic mechanical and rheological analysis revealed that the incorporation of PBS in CS/PLA-ADR-PBS composites led to a non-monotonic behaviour, with *G*′, *G*″, and complex viscosity initially increasing at low PBS contents (≈5–10%) and decreasing at higher loadings. At lower concentrations, PBS forms a crosslinked network with CS and PLA *via* ADR, whose epoxy groups undergo ring-opening reactions with amino, carboxyl, and hydroxyl groups, thereby enhancing interfacial compatibility. The improved network structure strengthened intermolecular interactions, which increased melt elasticity, energy dissipation, and resistance to flow (Fig. 9). The flexible PBS chains further enhanced cavitation and shear band formation during deformation which allowed the material to deform easily and absorb more energy (improving toughness). When the PBS content exceeded 15%, the excess flexible phase disrupted the network structure and weakened intermolecular interactions. As a result, the modulus and viscosity decreased. The excess PBS also acted as a lubricant, increasing chain mobility and reducing energy storage and flow resistance.

**Fig. 9:**
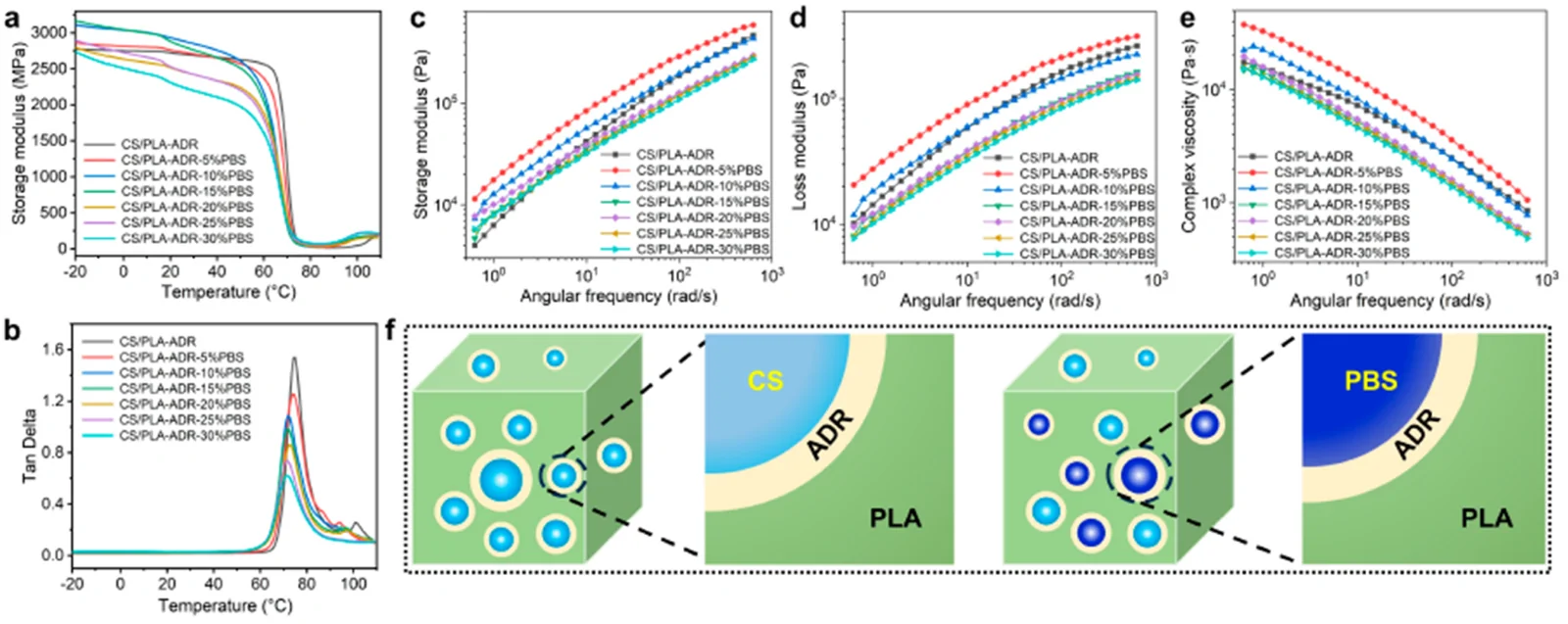
Rheological behaviour of CS/PLA-ADR-PBS composites with varying PBS content: (a) storage modulus *vs.* temperature; (b) loss angle, (c) storage modulus, (d) loss modulus, and (e) complex viscosity as a function of frequency; (f) schematic illustration of the compatibilization and toughening mechanism of PBS in CS/PLA-ADR-PBS composites. Reproduced under the terms of the Creative Commons Attribution (CC BY) License from ref. 34. Copyright 2025. MDPI Publisher.

The effect of PBS as a flexible phase on melt flow behaviour of PLA/PBS blends was further studied by Choi *et al.*^109^ The addition of PBS reduced the complex viscosity, indicating easier melt flow and higher chain mobility. Similarly, both *G*′ and *G*″ decreased with increasing PBS content, exhibiting reduced melt elasticity and weaker intermolecular interactions. The blends also exhibited stronger shear-thinning behaviour at higher PBS concentrations. Consistent with the findings of Yan *et al.*,^34^ phase separation and weaker interfacial adhesion at PBS content of approximately 10% or above were associated with reduced stress transfer and weaker viscoelastic response.

### 5.2 Reactive compatibilization and molecular architecture

The rheological behaviour of non-reactive biopolymer blends are mainly controlled by lubrication and free-volume effects. In contrast, that for reactive biopolymer blends, rheological properties are mostly defined by stronger intermolecular interactions and a stable melt structure. For example, Altinbay *et al.*^86^ investigated the rheological behaviour of PLA/PBAT blends at different processing temperatures using Joncryl as a chain extender. The addition of Joncryl significantly increased η* and *G*′ (Fig. 10(e) and (f)) by increasing molecular weight of the polymer chains and improved melt elasticity by chain extension and branching reactions. These effects became more apparent at higher processing temperatures. The high temperature enhanced reactivity and transesterification which enhanced PLA and PBAT compatibility. Joncryl improved the dispersion of PBAT by producing smaller domains. The larger interfacial area restricted polymer chain mobility, leading to better viscoelastic behaviour. In contrast, unmodified blends and aged-PBAT blends showed coarser and poorly dispersed domains. These coarse domains resulted in lower viscosity and weaker elastic behaviour of the material. Also, excessively high processing temperatures caused PLA degradation, which partially reduced the rheological performance.

**Fig. 10:**
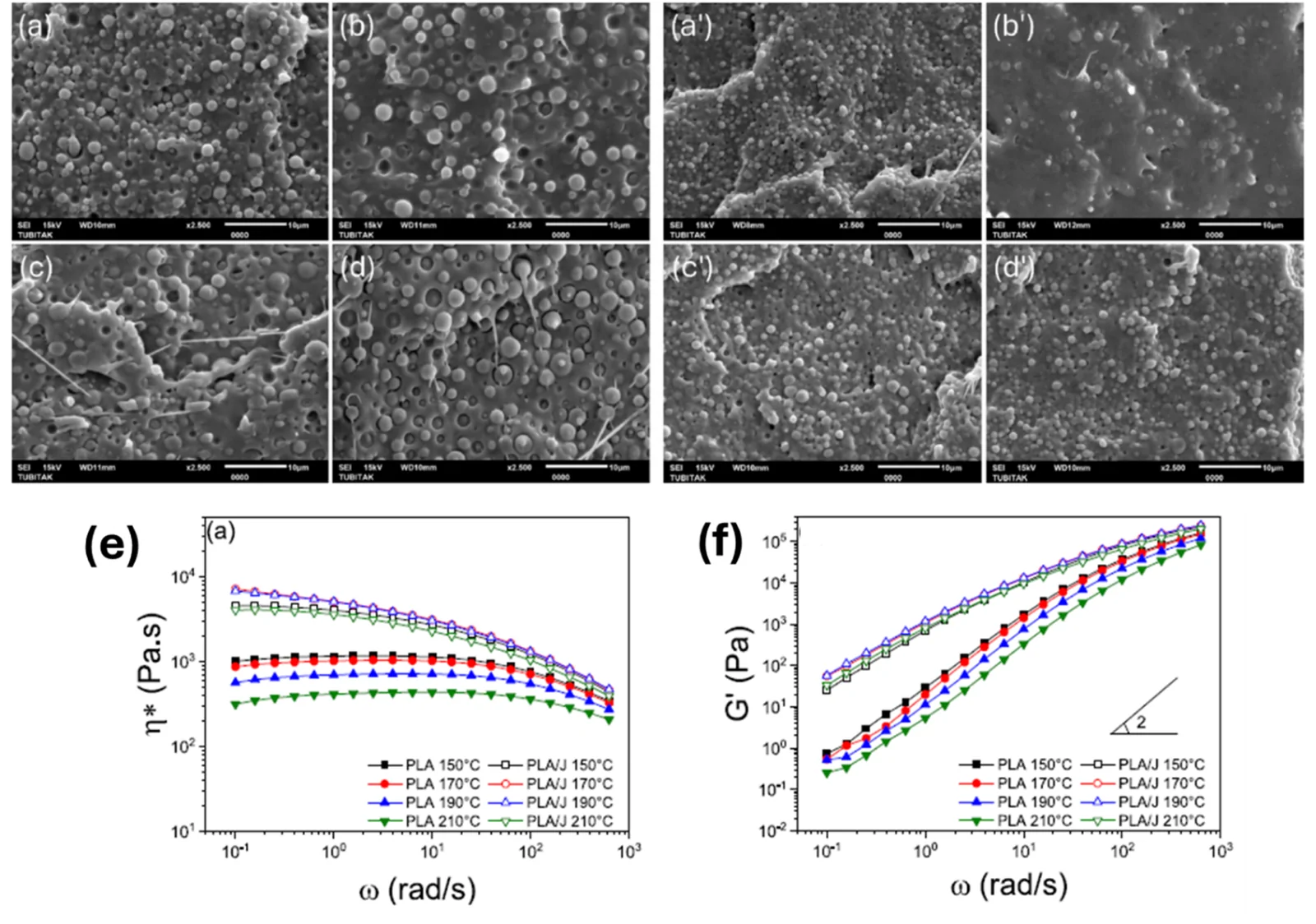
SEM micrographs of PLA/PBAT blends processed at 150 °C (a and a′), 170 °C (b and b′), 190 °C (c and c′), and 210 °C (d and d′): unmodified blends (a–d) and Joncryl-modified blends (a′–d′). (e) Complex viscosity (η*), and (f) storage modulus (*G*′) *vs.* frequency plots for uncompatibilized and Joncryl-compatibilized PLA samples prepared at different processing temperatures and measured at 190 °C. Reproduced with permission from ref. 86. Copyright 2025. Elsevier Science Ltd.

While Joncryl mainly improved melt elasticity through chain extension and branching, Wen *et al.*^76^ showed that graft compatibilizers can further enhance rheological behaviour through interfacial copolymer formation in PLA/PBAT blends. The addition of PLA-*g*-ECA significantly increased η* (Fig. 11(a)) and *G*′, especially at higher compatibilizer loadings (10 and 15 wt%; L/B/P/10LE and L/B/P/15LE). The blends also showed stronger shear-thinning behaviour and an enhanced elastic response, as observed from the Han plot analysis (Fig. 11(b)). These changes were due to the formation of grafted copolymers at the interface and increased chain entanglement between PLA and PBAT. The improved melt elasticity also resulted in a finer PBAT dispersion and stabilized the morphology during processing. However, excessively high compatibilizer contents resulted in smaller improvements due to structural saturation and phase coalescence.

**Fig. 11:**
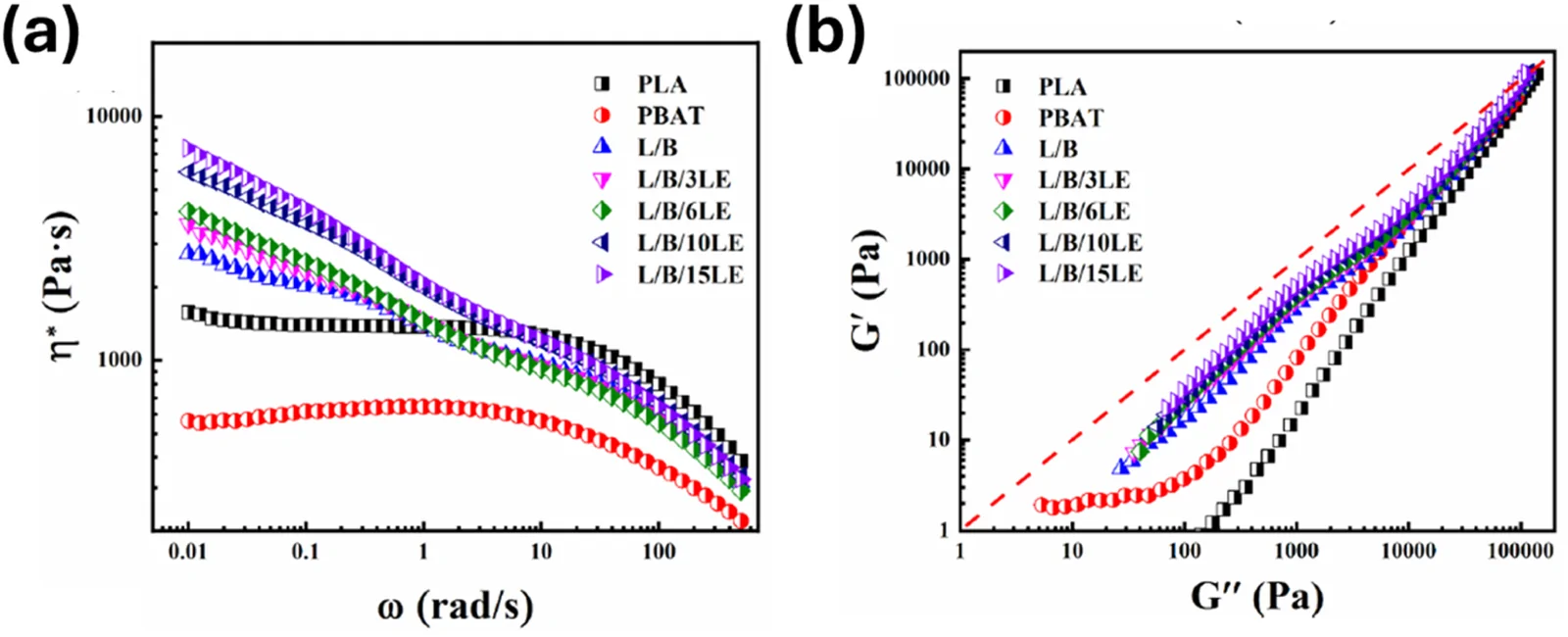
PLA/PBAT blends' (a) complex viscosity (η*) *versus* angular frequency and (b) Han plots of PLA/PBAT blends with varying PLA-*g*-ECA content. Reproduced with permission from ref. 76. Copyright 2024. American Chemical Society.

Ternary blends have greater morphological complexity, making interfacial interactions and adequate melt strength even more crucial during processing. Chuakhao *et al.*^57^ investigated the rheological behaviour of reactive and non-reactive PBS/PLA/PBAT blends. Although PBS improved the morphology of the non-reactive blends, these blends still had low viscosity and insufficient melt strength. This limited their suitability for processes such as blown film extrusion. Weak interfacial interactions allowed the polymer chains to move freely, resulting in poor viscoelastic behaviour. In contrast, the reactive blends had improved rheological properties because of stronger interfacial bonding and partial crosslinking. These interactions formed an interconnected structure that restricted polymer chain mobility which increased the complex viscosity and storage modulus. The blends also exhibited elastic behaviour (*G*′ > *G*″) at low frequencies due to the formation of a network-like melt structure. As a result, the reactive blends had higher melt strength and produced more stable bubbles during blown film extrusion. Notably, PBS improved morphology in the non-reactive systems, but reactive compatibilization was necessary to achieve the rheological properties required for industrial processing.

While reactive PBS/PLA/PBAT blends showed improved melt strength through partial crosslinking, Heidari *et al.*^110^ demonstrated that reactive compatibilizers such as Joncryl can further enhance melt elasticity and interfacial homogeneity in PLA/PBAT blends. The addition of Joncryl significantly increased the zero-shear viscosity and caused a ∼68% increase in torque, indicating chain extension and long-chain branching within the blend. These structural changes improved melt elasticity, as reflected by higher *G*′ values and a shift of the *G*′/*G*″ crossover to ∼2 rad s^−1^. This behaviour suggested slower droplet relaxation and stronger interfacial entanglement between the phases. In contrast, blends containing Lotader (another chain extender investigated in this study) exhibited lower viscosity and weaker viscoelastic response, consistent with less effective interfacial interaction.

Advanced reactive extrusion strategies have also demonstrated that controlled interfacial grafting can significantly enhance melt elasticity, relaxation behaviour, and processability in PLA/PBS blends. Chang *et al.*^111^ investigated the rheological behaviour of biopolymer blends modified *via* UV-induced reactive extrusion using glycidyl methacrylate (GMA) and trimethylolpropane triacrylate (TMPTA), demonstrating that interfacial grafting (Fig. 12(a)) and long-chain branching significantly alter melt viscoelastic properties. The introduction of GMA and TMPTA led to a pronounced increase in η* and *G*′, particularly at low frequencies, indicating enhanced molecular entanglement and the formation of long-chain branched structures. The blends exhibited typical shear-thinning behaviour. However, the upward deviation of η* with increasing GMA content reflected increased resistance to flow and improved melt strength (Fig. 12(b)). The Cole–Cole plots (Fig. 12(c)) became more circular after compatibilizer addition, indicating improved phase compatibility and more uniform relaxation behaviour. Longer relaxation times also suggested that polymer chain mobility was more restricted. These observations are due to the formation of graft copolymers at the PLA/PBS interface, which reduced interfacial tension and enhanced interfacial entanglement. Notably, at higher GMA contents (*e.g.*, G15T10 with 1.5 phr GMA and 1 phr TMPTA), a reduction in viscosity was attributed to a possible lubricating effect caused by over-saturation of reactive species. Overall, UV-induced reactive compatibilization can enhance melt elasticity and viscosity through controlled branching and interfacial coupling, thereby improving processability and structural stability of PLA/PBS blends, although the extent of improvement depends on the balance of interfacial coupling and extent of modification.

**Fig. 12:**
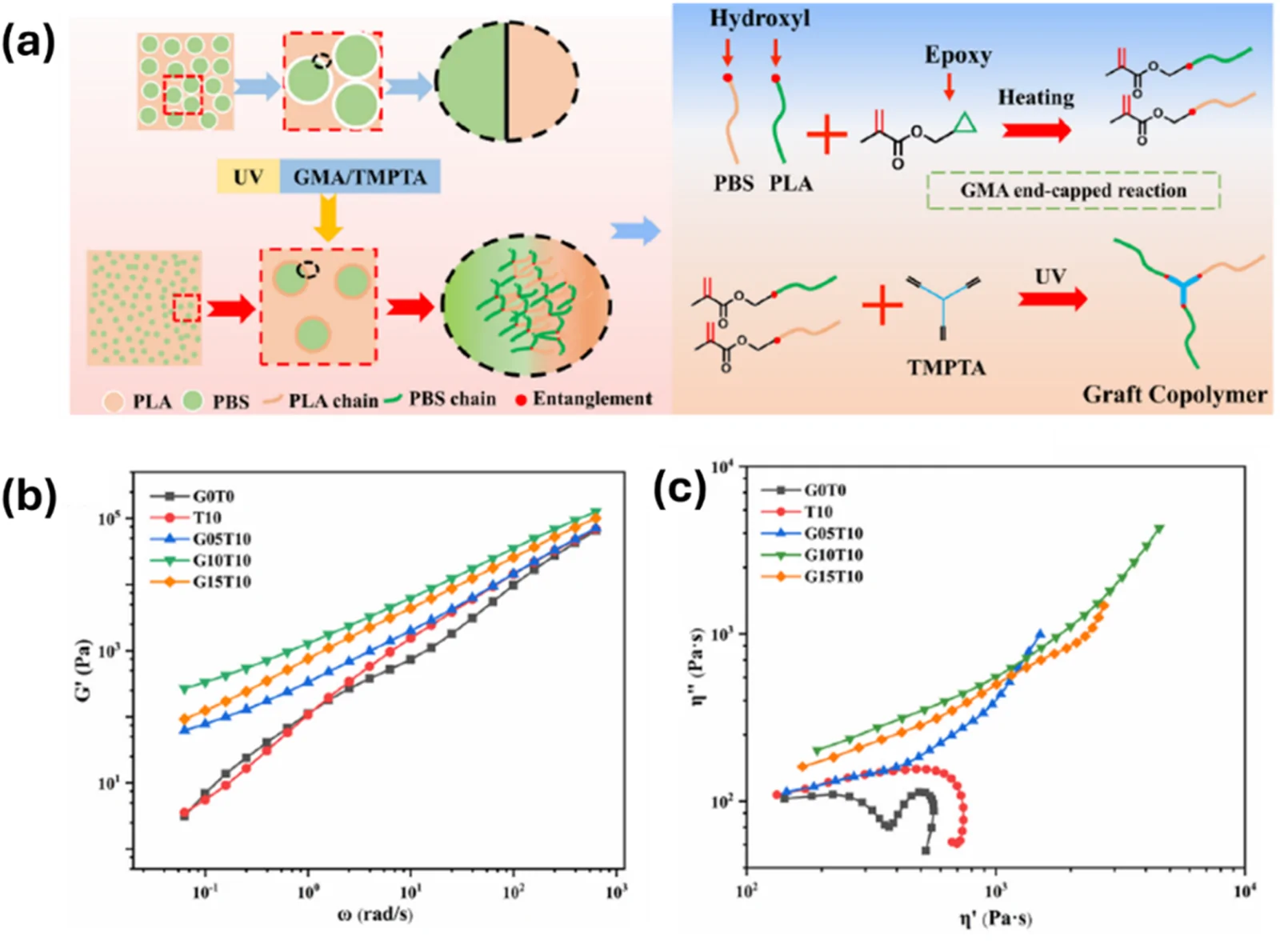
(a) Schematic illustration of compatibilization of PLA/PBS blends using GMA and TMPTA; dynamic rheological properties of PLA/PBS samples: (b) storage modulus (*G*′) and (c) complex viscosity (η*). Reproduced with permission from ref. 111. Copyright 2022. Elsevier Science Ltd.

### 5.3 Filler-induced network formation and melt reinforcement

More recent approaches to tailoring rheological behaviour have focused on incorporating nanoscale fillers capable of restricting chain mobility and promoting network formation within biodegradable matrices. However, these effects are not universal, they depend on filler characteristics and processing history. Delorme *et al.*^98^ observed the rheology of PBAT films modified with different wt% of SORBACID® 911 layered double hydroxide (LDH). The rheological behaviour of PBSA–LDH nanocomposites indicated that the incorporation of LDH fillers had minimal impact on melt viscoelastic properties, suggesting good processability of the system. Cole–Cole plots of neat PBSA and LDH-filled composites largely overlapped (Fig. 13), demonstrating that the addition of LDH did not significantly alter melt viscosity or chain relaxation dynamics at processing temperatures (∼110 °C). This behaviour implies that the LDH particles, despite acting as reinforcing fillers in the solid state, did not form a percolated network in the melt that would restrict polymer chain mobility. Consequently, the composites retained flow characteristics similar to those of neat PBSA, allowing them to be processed using conventional extrusion and blown film techniques. This rheological stability is important because it shows that the improvements in mechanical stiffness and barrier performance mainly resulted from the solid-state morphology, such as filler reinforcement and tortuous diffusion pathways, rather than changes in the melt structure. The absence of a significant increase in viscosity also suggests that the LDH was well dispersed and did not form large agglomerates that could disrupt melt flow.

**Fig. 13:**
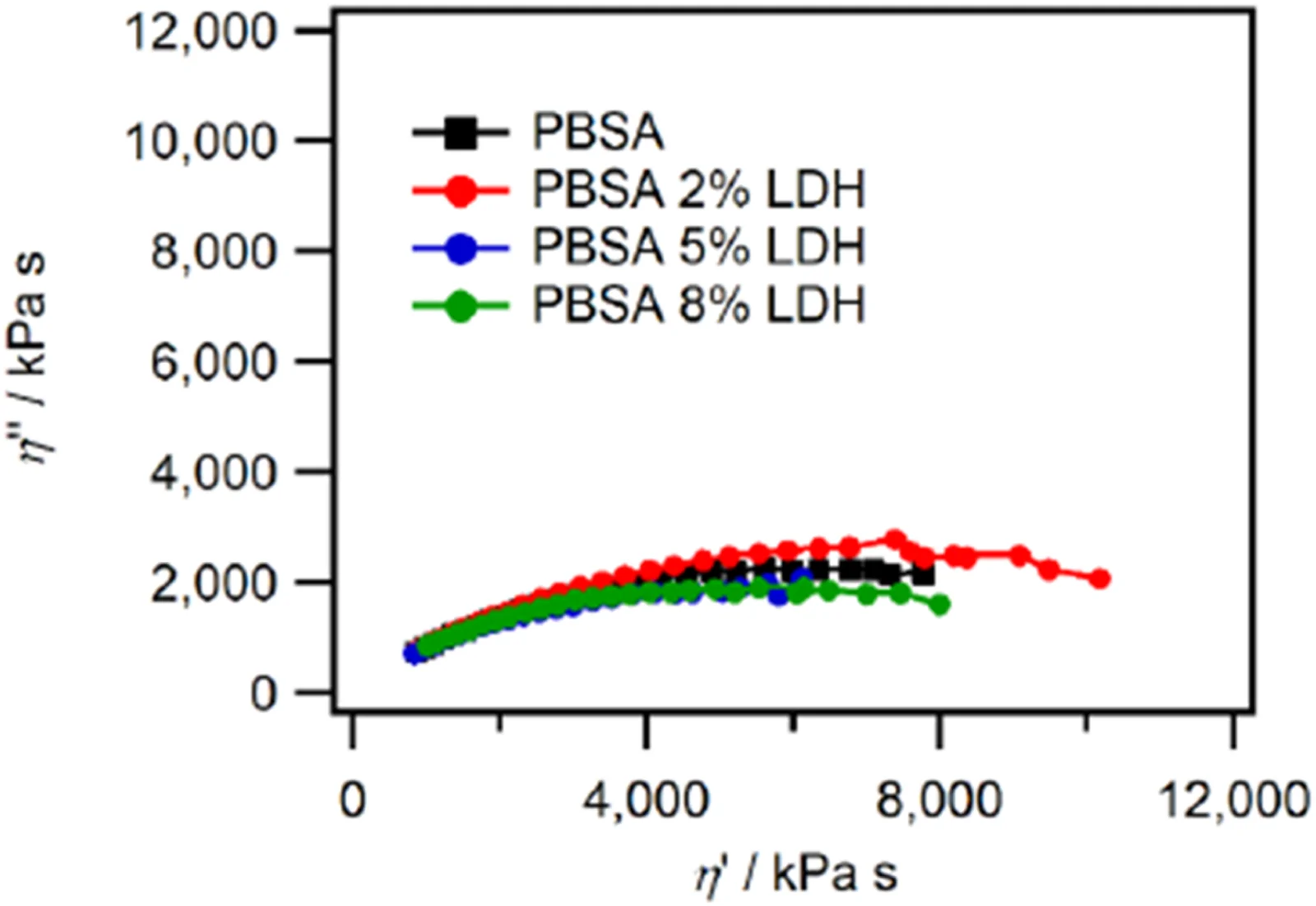
Cole–Cole plots of PBSA and PBSA/LDH nanocomposite films obtained from melt viscoelastic measurements at 110 °C. Reproduced under the terms of Creative Commons Attribution (CC BY) License from ref. 98. Copyright 2022. MDPI Publisher.

Better filler dispersion improves interfacial interactions, leading to interconnected melt structures and enhanced rheological properties in biopolymer nanocomposites. The rheological properties of biodegradable PBSA/PHBV blends have been modified by introducing surface-modified nanoclay with *N*-octadecylamine *via* two different processing routes.^101^ Nanoclay increased both the *G*′ and η* of the blend by improving polymer–filler interactions and restricting chain mobility. However, the extent of rheology enhancement was governed by the processing route. Nanocomposites prepared by one-step blending behaved like a pseudo-liquid because the nanoclay was poorly dispersed. The resulting agglomerates limited effective stress transfer in the melt. In contrast, the two-step masterbatch approach produced much better nanoclay dispersion. The exfoliated nanoclay was located at the PBSA/PHBV interface, leading to pseudo-solid or gel-like behaviour at higher nanoclay loadings. The better filler distribution led to the formation of a percolated network structure and a reduced percolation threshold.

Bio-based fillers such as microfibrillated cellulose (MFC) can alter the melt viscoelastic behaviour of PLA/PBSA blends. Apicella *et al.*^99^ examined the rheology of PLA/PBSA films modified with MFC and cardanol. All blends showed typical shear-thinning behaviour. As the shear rate increased, the viscosity decreased, which is beneficial for film processing. Cardanol also lowered the zero-shear viscosity (*η*_0_*) from approximately 6039 to 3581 Pa s by acting as a plasticizer and increasing polymer chain mobility. Unlike cardanol, MFC increased the viscosity and melt elasticity, particularly at 0.75 wt% loading (*η*_0_* ≈ 4595 Pa s). This improvement resulted from stronger polymer–filler interactions and better network formation, providing stable rheological behaviour for film blowing. A finer dispersion of MFC at intermediate concentrations enhanced viscoelastic response, while higher loadings (1 wt%) led to agglomeration, limiting further rheological improvement. In summary, the rheological properties of the blends were determined by the balance between plasticizer-induced chain mobility and nanofiller-induced network formation. Optimal filler dispersion provided both improved processability and better melt stability.

Further, Kostenko *et al.*^112^ demonstrated that the surface chemistry of functionalized nanoclays can substantially affect the elastic-dominated behaviour through stronger interfacial interactions and percolated filler networks. The rheological behaviour of PLA/PBAT nanocomposites modified with functionalized nanoclays, demonstrated that melt viscoelastic properties were highly sensitive to filler surface chemistry and dispersion quality. The incorporation of polymer-grafted clays, particularly poly(butyl acrylate) (PBA)-grafted systems, led to a pronounced increase in melt elasticity, with storage modulus reaching values on the order of ∼10^5^ Pa, and exceeding the loss modulus, indicating a transition toward elastic-dominated behaviour. The appearance of a secondary low-frequency plateau in *G*′ further confirmed the formation of a percolated filler–polymer network capable of effective stress transfer. In contrast, nanocomposites containing APTES-functionalized clays exhibited comparatively lower *G*′ and *G*″ values, attributed to reduced interfacial interactions and partial polymer degradation, which weakened the melt structure. η* showed typical shear-thinning behaviour in all systems, with the grafted-clay composites showing the highest viscosity and strongest resistance to flow. Cole–Cole and van Gurp–Palmen analyses indicated that the composites exhibited mostly homogeneous viscoelastic behaviour, although small deviations suggested minor structural heterogeneity in the modified systems.

The reinforcing effect of PLA fibres on the rheology of PBS composites was further investigated by Li *et al.*^113^ The melt viscoelastic properties of blend primarily depended on fiber content and network formation. The addition of PLA fibers significantly increased both η* and *G*′, indicating improved melt elasticity and reduced polymer chain mobility. Notably, viscosity increased dramatically from ∼1864 Pa s (neat PBS) to ∼43 000 Pa s at 30 wt% PLA fibers. This indicated the formation of a highly interconnected structure in the blend. A rheological percolation threshold occurred at approximately 17.5 wt% fibre content. Beyond this threshold, the fibres formed an interconnected network that changed the melt behaviour from viscous-dominated to more elastic. This transition was further supported by pronounced shear-thinning behaviour at PLA fibre contents of 20 wt% or higher. Deviations in the Cole–Cole plots also indicated structural heterogeneity and longer relaxation times associated with the fibre network. Overall, the formation of a continuous fiber network restricted polymer chain dynamics and enhanced stress transfer within the melt.

The incorporation of viscoelastic secondary phases has also been explored as a way to tune melt elasticity and flow behaviour in immiscible biodegradable blends. Zhang *et al.*^114^ studied the rheological behaviour of immiscible PBAT/poly(vinyl acetate) (PVAc) blends and found that the addition of PVAc significantly altered the viscoelastic response due to its higher viscosity and elasticity. *G*′ increased from ∼105 MPa for neat PBAT to ∼200 MPa at 30 wt% PVAc, indicating improved melt elasticity and resistance to deformation. The phase angle also decreased from ∼88° to ∼55°, showing a shift toward more elastic behaviour in the blends. Similarly, complex viscosity (η*) increased at low frequencies with increasing PVAc content, while all blends maintained typical shear-thinning behaviour, which is beneficial for processing (Fig. 14). In addition, tan *δ* decreased with PVAc addition, indicating lower viscous dissipation and faster elastic response. Despite the immiscible nature of the system, Cole–Cole analysis revealed smooth arcs, suggesting relatively uniform dispersion of PVAc within the PBAT matrix, although SEM visualized a “sea-island” morphology with increasing droplet size (from ∼0.56 µm to ∼2.51 µm) at higher PVAc loadings.

**Fig. 14:**
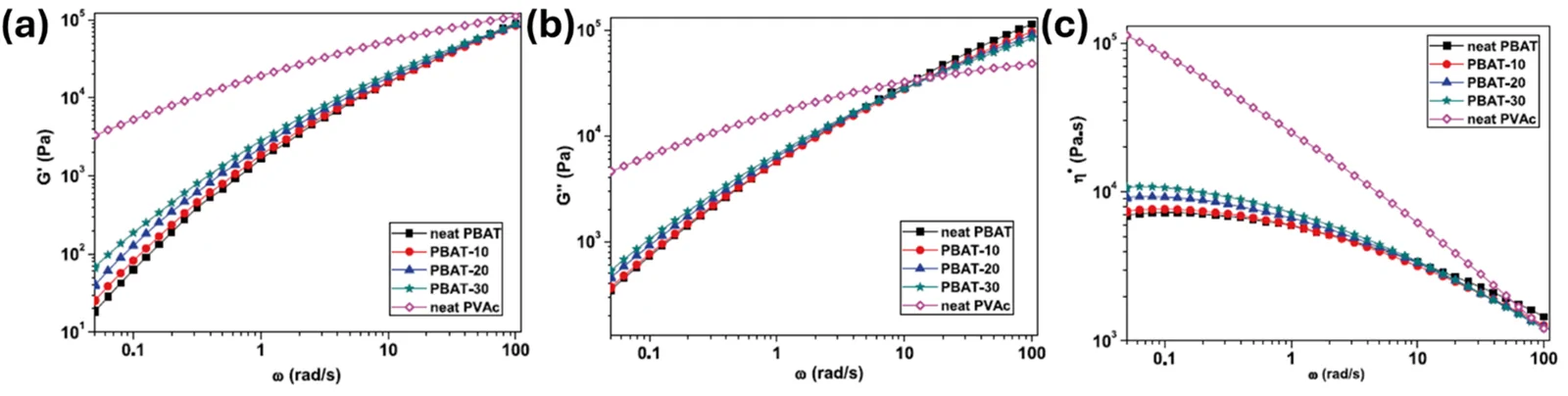
(a) Storage modulus (*G*′), (b) loss modulus (*G*″), and (c) complex viscosity (η*) as a function of angular frequency (*ω*) for neat PBAT, neat PVAc, and PBAT/PVAc blends. Reproduced with permission from ref. 114. Copyright 2022. Springer Nature.

Overall, the reviewed studies show that rheological modification is often non-linear. Increasing the content of a flexible phase, compatibilizer, or filler does not necessarily produce continuous improvement. Instead, the rheological response depends on reaching an appropriate balance between chain mobility, interfacial interactions, and structural development. Low levels of PBS or compatibilizers can enhance melt elasticity, whereas excessive addition may disrupt the structure or produce diminishing returns. Similarly, filler reinforcement depends strongly on dispersion and network formation, with agglomeration limiting the rheological benefit. These findings indicate that optimum modification, rather than maximum addition, is important for achieving the required melt response. Despite these advances, quantitative correlations between morphological features and rheological parameters remain limited. The combined effects of blend composition, reactive modification, filler incorporation, and processing history also require further attention in multiphase systems. The future studies should aim to establish more consistent links between rheological measurements and phase morphology and practical processing window to guide the design of biodegradable blends with reliable processability and performance.

## 6 Effect of blend modifications on biodegradability and degradation behaviour

Biodegradable polyesters such as PBS, PBSA, PBAT, and PHAs generally degrade through hydrolytic chain scission followed by microbial or enzymatic assimilation. In soil environments, degradation is governed by the availability of moisture and microbial activity, together with temperature, oxygen availability, pH, and soil characteristics. Under dry conditions, limited water availability can suppress hydrolytic chain scission and microbial activity, substantially slowing degradation.^115–117^ Water first penetrates the polymer matrix and cleaves susceptible ester bonds, reducing molecular weight and generating shorter oligomers and monomers. These products can then be further metabolized by microorganisms, ultimately forming CO_2_, water, biomass, and, under anaerobic conditions, CH_4_. The degradation rate depends not only on the chemical structure of the polymer but also on crystallinity, molecular weight, chain mobility, hydrophilicity, morphology, and accessibility of the ester groups.

In blends, degradation behaviour depends on the composition and phase morphology of the constituent polymers. Modifications introduced to improve mechanical, rheological, or barrier performance can therefore also alter biodegradation by changing the structure and morphology of the blend. Thus, their effects on biodegradation are not necessarily unidirectional. The following section first examines the effect of blend composition and phase morphology on degradation behaviour, followed by other modifications such as compatibilization and chain extension, and filler addition and processing conditions.

### 6.1 Effect of blend composition and phase morphology on degradation

van der Zee *et al.*^118^ demonstrated that blend composition can be used to tailor the disintegration behaviour of biodegradable polyesters (PHBV, PCL, PBS, and PBSA) in soil. For PHBV-based blends, increasing the PBSA content progressively increased surface discoloration, hole formation, and fragmentation during soil exposure. PHBV/PCL blends showed similar, even faster disintegration. Similarly, PBS underwent severe deterioration within 4 weeks and physical fragmentation within 112 days, while blending PBS with PBSA accelerated fragmentation to approximately 8 weeks. The study further demonstrated that the degradation of PBS was highly dependent on the soil environment, as the use of biologically active compost inoculum substantially accelerated disintegration compared with uninoculated soil. For PBS/PBSA blends, the molecular weight decreased from 157 to 122 kg mol^−1^ for 75/25, 159 to 118 kg mol^−1^ for 50/50, and 157 to 122 kg mol^−1^ for 25/75 blends after 112 days. However, these changes should not be directly interpreted as biodegradation because similar molecular-weight reductions were observed in abiotic controls and the authors attributed them primarily to polymer ageing and selective recovery of less-degraded material. In PLA-based systems, 20 wt% PBSA did not produce visible deterioration even after 336 days, whereas the PLA/PBSA 50/50 blend showed a rapid loss of its initial strain at break of 215 ± 123% within the first 25 days, together with a progressive decrease in tensile strength. In contrast, PLA/PCL 50/50 showed substantial disintegration, demonstrating a stronger effect of PCL incorporation on the degradation behaviour of PLA. Importantly, CO_2_ evolution measurements confirmed that the effect of blending was not limited to physical disintegration. The PLA/PCL 80/20 blend achieved approximately 39% conversion of polymer carbon to CO_2_ after 12 months in soil and 89% after 6 months under home-composting conditions. Taken together, these results suggest that the composition-dependent degradation behaviour arises from changes in the accessibility (to enzymes and microorganisms) and continuity of the individual polymer phases, which can influence the extent to which water, microorganisms, and degradation-active species reach the polymer matrix. This is an inference from the observed composition-dependent disintegration and mineralization behaviour, rather than a mechanism directly established by van der Zee *et al.*^118^ The study shows that blending can change both the physical disintegration rate and, in systems such as PLA/PCL, the extent of biodegradation. However, the effect varies with the degradation environment.

The influence of composition on biodegradation is also reported for blends of PLA with PBS, PBAT, and TPS. Ludwiczak *et al.*^119^ prepared PLA-based blends containing 30, 50, and 70 wt% of PBS, PBAT, or TPS and evaluated their disintegration in synthetic compost for 65 days. The neat polymers showed degrees of disintegration of 97% for PLA, 99% for PBS, 96% for PBAT, and 100% for TPS. The degradation behaviour of the blends depended on the polymer pair and composition. Previous studies cited by the authors showed that PLA/TPS blends generally degraded more rapidly than PLA/PBS blends, whereas PLA/PBAT blends could exhibit slower degradation than the corresponding neat polymers. This difference can be related to the accessibility of the polymer chains to hydrolysis and enzymatic attack. PLA degradation occurs mainly through hydrolysis of ester bonds, followed by microbial uptake of the lower-molecular-weight products. However, the rate of this process depends on factors such as crystallinity, molecular weight, and *T*_g_. The amorphous regions are more accessible to water and enzymes, whereas the compact crystalline regions restrict their penetration. Therefore, incorporation of a second polymer can change the fraction and accessibility of amorphous regions, as well as the continuity of the individual phases. In PLA/TPS blends, the presence of the more readily degradable starch phase can facilitate degradation and increase the accessible regions within the blend. In contrast, a less accessible or slower-degrading second phase can limit the exposure of the PLA matrix and delay overall disintegration. Thus, blending does not necessarily accelerate biodegradation. The effect depends on how the second phase changes the morphology, crystallinity, and accessibility of the polymer matrix to water and microorganisms.

Evidently, role of composition can also be linked to changes in crystallinity. Sabalina *et al.*^120^ investigated PBS/PLA and PBS/PHB blends at 70/30 and 50/50 ratios during six months of immersion in artificial seawater. The PBS/PHB 50/50 and 70/30 blends showed matrix-droplet and co-continuous morphologies, respectively. The PBS crystallinity decreased from 51.2% for neat PBS to 34.8% for the 50/50 PBS/PHB blend and 30.8% for the 70/30 blend. Such changes in crystallinity are relevant, because as stated above, the crystalline regions restrict water diffusion, whereas the amorphous regions are more accessible to hydrolytic degradation. Thus, composition-induced changes in crystalline-amorphous structure can modify the accessibility of the polymer to the degradation medium. The maximum water uptake of the vertically printed PBS/PHB blends was 2.12 ± 0.20% for the 50/50 blend and 1.82 ± 0.07% for the 70/30 blend, while the corresponding PBS/PLA blends showed values of 1.53 ± 0.15% and 2.34 ± 0.25%, respectively. The higher water uptake of some 70/30 samples was not explained solely by composition. The poorer processability and a higher concentration of printing-induced voids provided additional pathways for water penetration. The degradation-related water uptake and leaching parameters also changed with composition and printing orientation, demonstrating that composition and morphology can jointly influence hydrolytic degradation.

This relationship between composition, morphology, and degradation is important because the most degradable polymer is not necessarily the phase that controls the overall degradation behaviour of the blend. In the PLA/PCL system. For example, the degradation of a dispersed PCL phase can be restricted by the surrounding PLA matrix. Similarly, the PBS/PBSA results show that increasing the PBSA fraction progressively accelerates disintegration, whereas the molecular-weight data of the recovered material do not directly reflect this trend because extensively degraded material is removed from the measurable fraction. Therefore, the effect of composition should be considered together with phase continuity, interfacial accessibility, crystallinity, and water transport in the blend.

### 6.2 Effect of compatibilizers and chain extenders

Depending on the chemistry of the modifier and the morphology generated, chain extension and compatibilization can either slow down degradation or accelerate it. This distinction is important because the structural changes that improve mechanical strength and processability do not necessarily have the same effect on biodegradation.

Luo *et al.*^121^ examined epoxidized cardanol oleate (ECD-OA) as a bio-based chain extender for PLA. ECD-OA reacted with PLA end groups and increased its molecular weight from 15.3 × 10^4^ to 17.1 × 10^4^ g mol^−1^ at 1 phr, while its long alkyl chains increased molecular entanglement and reduced melt flow. The long-chain structure of ECD-OA increased chain entanglement and disrupted the regular arrangement of PLA chains. During proteinase-K degradation, the modified films nevertheless showed greater mass loss than neat PLA. After 96 h, PLA/1ECD-OA exhibited 68.4% mass loss, compared with the lower mass loss of neat PLA, while the commercial NC514 chain extender produced 73.9% mass loss. The modified films also developed extensive surface holes and uneven degradation compared with the relatively intact surface of neat PLA. The bio-based and relatively small molecular structure of the chain extender enhanced the biodegradation. However, the long-branched structure of ECD-OA also produced greater chain entanglement and slower degradation than NC514. This illustrates a more complex trade-off. Chain extension increased molecular weight and improved mechanical and thermal performance, while the chemical nature and architecture of the modifier determined how strongly these changes affected degradation. The result also suggests that bio-based chain extension does not necessarily sacrifice biodegradability, provided that the structure of the chain extender remains susceptible to the degradation process.

Reactive compatibilization of PLA/PBAT (70/30) using poly(propylene glycol) diglycidyl ether (PPGDGE) was reported by Gao *et al.*^122^ The epoxy groups of PPGDGE reacted with the hydroxyl and carboxyl end groups of PLA and PBAT, producing PLA-*g*-PBAT copolymers *in situ* at the interface. This reduced the phase separation between the two polymers. At 5 phr PPGDGE, the PBAT dispersed phase showed the smallest and most uniformly distributed domains, while AFM revealed a smoother and more continuous phase structure. This morphological refinement was accompanied by a fourfold increase in elongation at break and a 3.5-fold increase in notched impact strength compared with the uncompatibilized blend. However, unlike conventional chain extension, the improved interfacial structure did not slow down degradation. Under hydrothermal conditions, increasing PPGDGE content progressively increased the mass-loss rate, with the magnitude of the fitted rate constant increasing from 0.5711 for the unmodified blend to 0.8582 at 6 phr PPGDGE. This behaviour was due to the hydrophilic polyether segments of PPGDGE, which promoted water penetration into the PLA/PBAT matrix and enhanced ester-bond hydrolysis. The degraded samples developed whitening, surface gullies, and progressive fragmentation. Thus, in this system, the formation of a finer and more continuous interfacial morphology improved mechanical performance without compromising hydrolytic degradation. Instead, the chemical structure of the compatibilizer introduced an additional pathway for water transport. Thus, the improved compatibility does not inherently lead to slower degradation. The effect depends on whether the compatibilizer restricts chain accessibility or facilitates penetration of the degrading medium.

Feijoo *et al.*^123^ examined the effect of the epoxy-functionalized chain extender Joncryl® ADR-4468 on PHBV/PBSA blends containing 30–70 wt% PHBV. Before modification, the blends exhibited clear phase separation. At 30 wt% PBSA, PBSA existed as a dispersed phase within PHBV, while increasing PBSA content led toward interconnected and co-continuous structures. Addition of 0.3–0.5 phr Joncryl did not change the overall morphology type, but it reduced the size of the dispersed domains and reduced interfacial voids. In the 50/50 blend, the modified material showed a smoother and more homogeneous morphology, with smaller interfacial gaps at the higher Joncryl content. The authors proposed that reactions between the epoxy groups and the polymer end groups generated PHBV-Joncryl-PBSA linkages, which reduced interfacial tension and improved stress transfer. The finer dispersion and stronger interface improved elongation and flexural behaviour, while chain extension and branching also increased melt elasticity and reduced the difference in viscosity between the two phases. Importantly, however, the study did not directly evaluate the biodegradation of the Joncryl-modified PHBV/PBSA blends. Therefore, the improved morphology and mechanical performance cannot be directly linked to a change in biodegradability. The authors instead cite earlier work in which peroxide-induced crosslinking of PHBV/PBS blends did not affect compostability.^124^ This distinction is important because reduced domain size and stronger interfacial bonding could either increase degradation by increasing interfacial area and facilitating water or microbial access, or slow down the degradation by restricting chain mobility and forming a more strongly connected network.

Reactive chain extension can improve the durability of a blend by strengthening the interface and reducing the accessibility of the polymer phases to the degradation medium. This behaviour was observed in PBAT/PLA blends compatibilized with an epoxy-based chain extender (CE).^125^ The CE promoted reactions with the terminal groups of PBAT and PLA, resulting in improved interfacial compatibility and a finer phase morphology with smaller dispersed PLA domains. The compatibilized PBAT/PLA (80/20/1 phr CE) blend showed a slower weight loss during 12 weeks of soil burial than the corresponding uncompatibilized blend, which exhibited a degradation trend similar to PBAT. At the pilot scale, the optimized film showed approximately 2.9% weight loss after three months of soil burial. Surface analysis further showed the development of scratches and degradation features with increasing burial time.

Compatibilization does not necessarily suppress degradation even when it produces stronger interfacial bonding. In PLA/chitosan (CS) composites, the epoxy chain extender ADR4468 reacted with hydroxyl and carboxyl groups of the two components and induced chemical linkages which formed a more stable interface.^126^ The modification reduced interfacial defects and promoted a more uniform dispersion of CS within the PLA matrix. Despite this increased interfacial bonding, the ADR-containing composites retained substantial degradation under composting conditions. After 100 days, the mass loss was 44.27–44.47% for composites containing 0.5–1.0% ADR, compared with 42.69% for the unmodified PLA/CS composite and 45.86% for neat PLA. Thus, the chain extender produced only a relatively small change in the overall degradation rate. The slightly higher degradation of the ADR-containing composites may be due to the improved bonding between PLA and CS, which allowed degradation of the CS phase to occur together with the surrounding PLA matrix.

Jeepery *et al.*^79^ investigated the enzymatic degradation of PHBH/PCL blends containing an atactic PHB-*b*-PCL diblock copolymer, using PHB depolymerase from *Rhodococcus pickettii* T1. The addition of the block copolymer reduced the enzymatic degradation rate in both ternary PHBH/PCL/BCP blends and binary PHBH/BCP blends. This was due to the reduction of the PCL segments, which are less susceptible to the enzyme and may restrict its access to hydrolysable PHBH chains. The block copolymer also improved phase dispersion and, depending on blend composition, enhanced flexibility and elongation at break. For example, the 80/20 PHBH/BCP blend exhibited an elongation at break of 480%, compared with 7.7% for neat PHBH, although its maximum stress decreased from 27.4 to 17.8 MPa. These results show that the block copolymer improves flexibility and blend morphology but slows enzymatic degradation. However, this effect was observed under specific enzyme test conditions and does not necessarily indicate slower degradation during composting or in the environment.

The reported effects of compatibilization and chain extension on biodegradation vary across polymer systems. Reactive modification often improves interfacial adhesion and refines phase morphology, but its effect on degradation is not uniform. In PBAT/PLA blends, chain extension produced a finer and stronger interface and slowed weight loss during soil burial. In contrast, ADR-modified PLA/chitosan showed similar mass loss to the unmodified blend despite stronger interfacial bonding. Thus, interfacial strengthening alone does not determine degradation behaviour. Modifier chemistry, phase morphology, crystallinity, chain mobility, and polymer accessibility to water and microorganisms all contribute. Moreover, studies use different measures, such as weight loss, disintegration, and CO_2_ evolution, under different conditions. These measures are not directly comparable. Therefore, the link between improved mechanical or rheological performance and long-term biodegradation remains poorly understood.

### 6.3 Effect of fillers and processing conditions

Effect of adding nanoparticles on the biodegradability of the blend depends on the type of nanoparticle, its interaction with the polymer, dispersion, and the resulting changes in crystallinity and water accessibility. Thus, nanoparticles that improve mechanical or barrier properties can either preserve or slow down degradation depending on how they modify the polymer structure.

The retention of biodegradability was demonstrated in PLA/PHBV blends reinforced with carbon nanomaterials. Ribeiro dos Anjos *et al.*^127^ incorporated multi-walled carbon nanotubes (MWCNT), graphene nanoplatelets (GNP), and their hybrids into an 80/20 PLA/PHBV blend. The biodegradation was evaluated from CO_2_ evolution over 150 days. Although the initial degradation rate varied between the formulations, all samples showed a similar overall trend and reached approximately 45% biodegradation after 150 days. Therefore, the carbon nanofillers did not substantially compromise the biodegradability of the blend. The faster initial degradation observed for the H1/3 MWCNT/GNP hybrid was related to differences in crystallinity, since crystalline regions are less accessible to hydrolysis and enzymatic attack than amorphous regions. This result is important because the nanofillers also improved the mechanical and antimicrobial properties of the blend, indicating that reinforcement does not necessarily require a loss of biodegradability.

The behaviour of cellulose-based nanoparticles provides further evidence that the degradation response is dependent on filler chemistry and morphology. The extensive review by da Costa *et al.* reports that cellulose nanocrystals (CNCs) can reinforce PLA and PBAT without necessarily reducing their biodegradability.^128^ In PBAT/CNC systems, both untreated and alkali-pretreated CNCs produced degradation rates similar to neat PBAT. Similarly, chemically modified CNCs increased the elastic modulus of PBAT and reduced water-vapor permeability, while biodegradation remained largely unaffected even after chemical modification. In contrast, adding 15 wt% CNC–ZnO hybrids to PLA increased soil-burial weight loss from about 8% for neat PLA to approximately 28% after 110 days. This behaviour was due to the hydrophilic nature of CNC, which facilitates water uptake into the otherwise hydrophobic PLA matrix. These results show that the effect of a nanofiller cannot be predicted simply from its reinforcement capability, its surface chemistry and influence on water transport are also important.

The opposite trend was obtained with eggshell powder (ESP), where improved barrier properties were accompanied by slower soil degradation. Li *et al.*^129^ incorporated 10–50 wt% ESP into potato starch/gelatin films. The particles were well dispersed at appropriate concentrations and formed hydrogen-bonding interactions with the starch and gelatin matrix. These interactions reduced water uptake and improved oxygen and water-vapor barrier properties. At 50 wt% ESP, water absorption after 60 min decreased from 272.46% for the unfilled film to 101.85%. The same reduction in water accessibility was reflected in the degradation behaviour. After 5 days of soil burial, the neat PS/G film had lost 73.61% of its mass, whereas the 50% ESP composite had lost only 50.24%. After 30 days, the 50% ESP film retained about 9.44% of its initial mass, while the unfilled and low-filler films were almost completely degraded. The increased hydrophobicity of the composite and the reduced penetration of water and microorganisms slowed the biodegradation.

Taken together, these studies show that nanoparticle addition can preserve, accelerate, or slow degradation depending on the structure produced by the filler. Carbon nanofillers in PLA/PHBV largely preserved CO_2_-based biodegradation, while hydrophilic CNC increased the degradation of PLA in one system. In contrast, ESP reduced water accessibility and slowed soil-burial mass loss. Reactive nanoparticles such as MuEpPOSS can further complicate this relationship by simultaneously reducing phase-domain size, increasing interfacial bonding, and restricting chain mobility, although their direct effect on biodegradation remains insufficiently studied. The review by da Costa *et al.*^128^ also highlights that nanoparticle location within the matrix, dispersed phase, or interface can substantially affect the resulting blend properties.

A key gap is therefore the lack of a quantitative structure–biodegradation relationship for nanocomposites. Most studies report filler loading together with mass loss or CO_2_ evolution, but fewer directly relate degradation to nanoparticle dispersion, interfacial area, crystallinity, water uptake, or phase localization. This is particularly important because the same structural changes that improve mechanical strength and barrier performance can also reduce the accessibility of polymer chains to water and microorganisms. Moreover, the available studies use different degradation measures and environments, making direct comparison difficult. Future work should therefore connect nanoparticle chemistry and localization with morphology, water transport, crystallinity, and degradation kinetics to identify filler designs that provide reinforcement without compromising end-of-life degradation.

## 7 Applications with enhanced functionalities

The control of composition, morphology, interfaces, and filler dispersion discussed above provides a basis for introducing additional functionalities into biodegradable polymer blends. These tailored structures have been explored for applications including antimicrobial and active food packaging, self-cleaning surfaces, UV-resistant agricultural films, biomedical materials, and additive manufacturing.

### 7.1 Additive manufacturing – 3D printing

Additive manufacturing, particularly fused filament fabrication (FFF), has often selected as a leading choice for processing biodegradable polymers due to its design flexibility and material efficiency. However, the performance of FFF-printed parts is highly dependent on rheology, interlayer adhesion, and processing conditions, which govern filament bonding, structural integrity, and mechanical anisotropy. Material modifications such as fibre reinforcement and reactive compatibilization further influence these properties, making the optimization of process–structure–property relationships essential for achieving high-performance printed components.

Donitz *et al.*^85^ reported the additive manufacturing, particularly FFF (Fig. 15(a)), and its effect on the mechanical performance of PBS-based composites reinforced with short (SF) and long (LF) hemp fibres. Fused filament fabrication and its effect on the mechanical performance of the material is governed by both material design and processing conditions. The incorporation of hemp fibres (SF and LF) significantly enhanced stiffness of the PBS composite, with the EM increasing from 510 MPa (PBS) to ∼820–840 MPa (Fig. 15(b)), but at the expense of tensile strength, which decreased from 35.5 MPa to ∼24–27 MPa due to stress concentration and interfacial limitations. This trade-off is also strongly affected by interlayer adhesion, which is a key factor in 3D printing. Processing modifications such as overlap (OL) and post-print heat treatment (HT) help reduce these limitations. Increasing the overlap between deposited filaments improves interfacial contact and reduces void formation, resulting in better load transfer and moderate improvements in strength and stiffness. Similarly, heat treatment promotes polymer chain diffusion across filament interfaces and relieves residual stresses, further improving structural integrity. As a result, the combined effect of OL and HT partially recovered the strength loss caused by fibre addition while maintaining the stiffness improvement. These findings highlight the importance of processing conditions in controlling the structure and final properties of 3D-printed PBS-based composites.

**Fig. 15:**
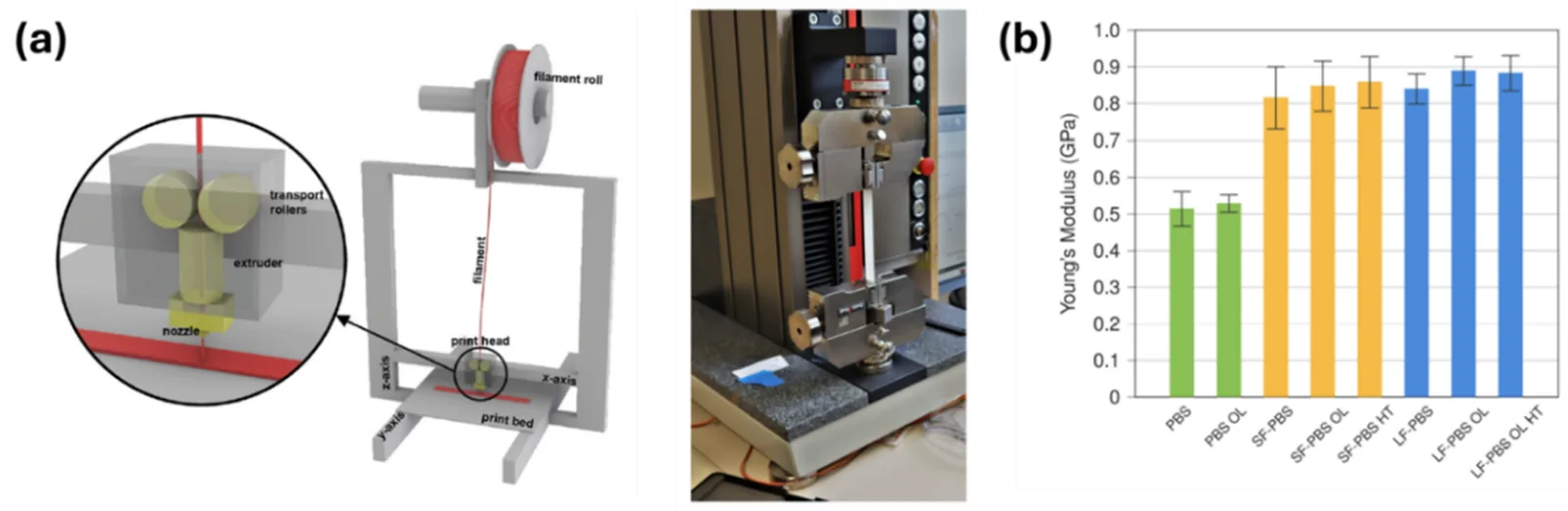
(a) Schematic of FFF process in Prusa i3MK3s 3D printer (b) Young's modulus of PBS composites with and without Hemp reinforcement and processing treatments. Reproduced under the terms of the Creative Commons Attribution (CC BY) License from ref. 85. Copyright 2023. MDPI Publisher.

Heidari *et al.*^110^ further demonstrated the suitability of optimized PLA/PBAT blends for FFF 3D printing and highlighted the important role of composition and reactive compatibilization in improving print performance. The addition of Joncryl not only improved melt rheology but also enhanced interlayer adhesion and the structural integrity of the printed parts. Under optimized conditions (15 wt% PBAT, 1.5 phr Joncryl, and 5 min residence time), the blends showed excellent mechanical performance, with tensile strength reaching ∼102 MPa, even higher than that of conventionally processed hot-pressed samples. The % EAB also increased significantly, reaching ∼66% in the 3D-printed PLA/PBAT-Joncryl blends, which corresponds to nearly a 1000% improvement over neat PLA. Similarly, toughness increased by more than 1300% (∼49.6 MPa). These improvements were attributed to better melt flow, stronger interfacial bonding, and the formation of microchannel structures during printing, which helped distribute stress and restrict crack propagation. Overall, the study showed that controlling rheology and compatibilization is essential for developing high-performance biodegradable materials for additive manufacturing.

### 7.2 Self-cleaning functionality

Self-cleaning biodegradable materials have attracted increasing interest, especially for packaging and coating applications where surface contamination is a major concern. These materials usually rely on photocatalytic nanoparticles that generate reactive species under light exposure to degrade organic contaminants. Their performance depends on nanoparticle activity, dispersion, and interactions with the polymer matrix.

Mtibe *et al.*^84^ investigated the self-cleaning behaviour of PBS/PBAT hybrid composites reinforced with lignin and ZnO nanoparticles. Photodegradation experiments using methylene blue showed that the neat PBS/PBAT blend exhibited almost no photocatalytic activity, with little change in dye concentration after 2 h of UV exposure (Fig. 16(a)). In contrast, the composites containing lignin and ZnO nanoparticles showed higher photocatalytic performance by attaining 70% dye removal. This improvement was due to the ZnO nanoparticles acting as active photocatalytic sites and improved dispersion and light absorption by lignin. The hybrid composite decolourized the dye solution, making it nearly colourless, while the control sample remained blue (Fig. 16(c)). This work demonstrated the potential of PBS/PBAT-based hybrid composites for self-cleaning surfaces and coatings.

**Fig. 16:**
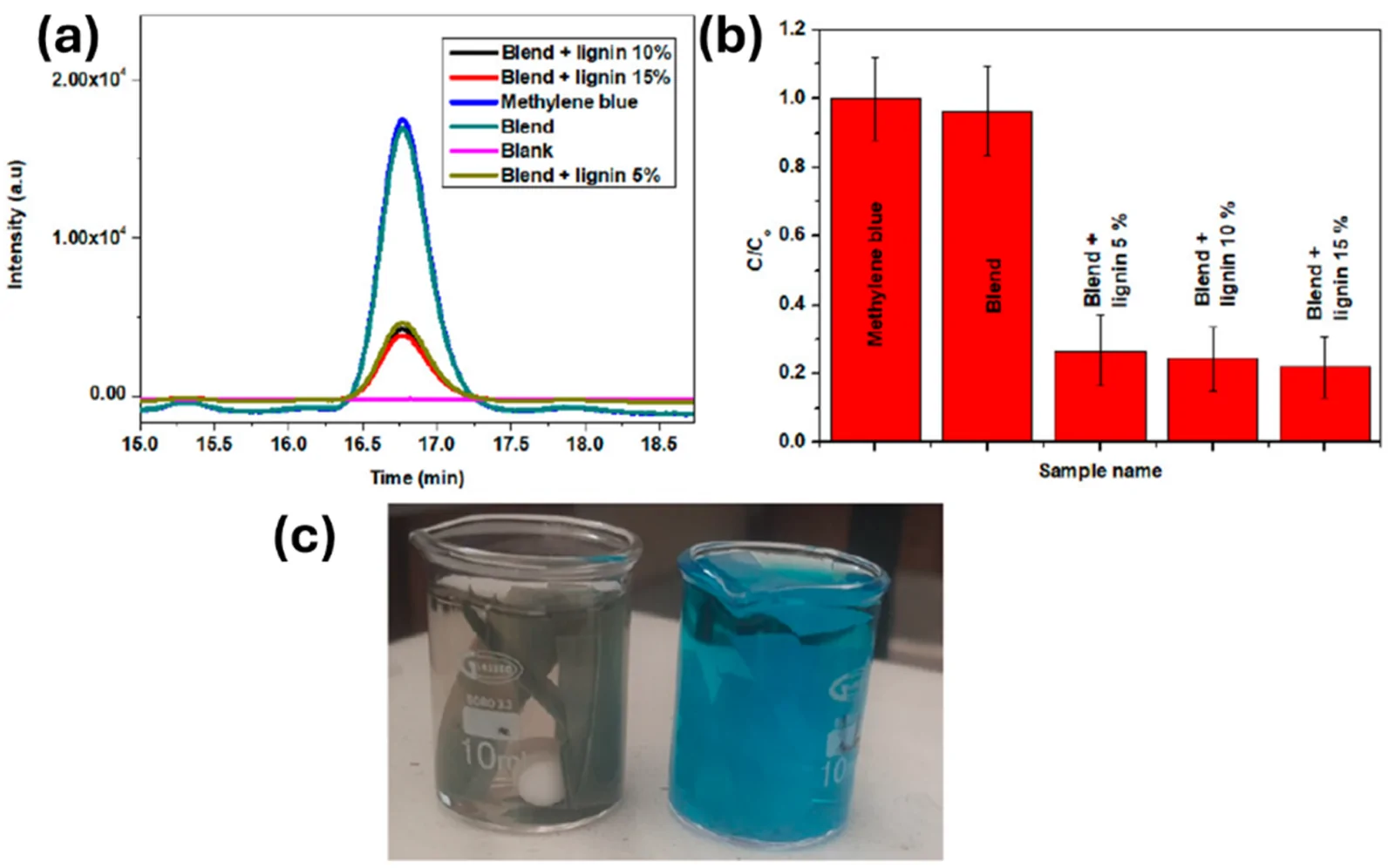
(a) Chromatogram of methylene blue; (b) photocatalytic efficiency of the materials; and (c) methylene blue solutions after 2 h of photodegradation using (left) blend with 15% lignin and (right) neat blend. Reproduced under the terms of the Creative Commons Attribution (CC BY) License from ref. 84. Copyright 2022. MDPI Publisher.

### 7.3 Antimicrobial functionality

Antimicrobial functionality of biodegradable polymer blends are relevant for food packaging and biomedical materials. This functionality can be introduced in the blends by adding active agents that inhibit bacterial growth through reactive oxygen species generation, membrane disruption, or ion release. The antimicrobial performance of the blends primarily depends on filler type, loading, dispersion, and interactions between the polymer–filler interactions.

Mtibe *et al.*^84^ also evaluated the antimicrobial performance of PBS/PBAT hybrid composites reinforced with lignin and ZnO nanoparticles against *E. coli* using the shake flask method (ASTM E2149-10). The neat blend showed significant bacterial growth, indicating no inherent antibacterial activity. In contrast, the hybrid composites completely inhibited *E. coli* growth due to the antibacterial properties of ZnO nanoparticles. An effective antimicrobial performance was achieved at a relatively low ZnO loading of 1.5 wt%. In addition, lignin may have contributed synergistically because of its intrinsic antimicrobial activity against both Gram-negative and Gram-positive bacteria.

### 7.4 Food preservation functionality

Biodegradable films with improved barrier and surface properties are essential for fresh food preservation. Packaging for food preservation requires controlled moisture loss and spoilage. Incorporating functional fillers allows biopolymer blends to better maintain product quality during storage.

Xue *et al.*^130^ validated the effectiveness of octadecylamine-modified cellulose (OMCC)/PBAT composite films through storage studies on strawberries, supported by both visual observations and quantitative weight loss analysis. The highest level of surface-modified OMCC (3#OMCC/PBAT films) provided the best preservation, keeping strawberries in good visual condition after 7 days with minimal decay and microbial growth (Fig. 17(a)). In comparison, the control and neat PBAT samples showed extensive spoilage. The MCC/PBAT films also reduced deterioration but were less effective than OMCC/PBAT films, highlighting the importance of surface modification. Weight loss measurements supported these observations (Fig. 17(b)). After 7 days, the control samples showed the highest weight loss (35–37%), followed by PBAT (30–32%). In comparison, MCC/PBAT and 3#OMCC/PBAT films had weight loss lowered to 18–20% and 15–17%, respectively, due to improved moisture retention. This improved preservation was attributed to the enhanced barrier properties of the films. OMCC created more tortuous pathways that slowed water vapour diffusion and reduced moisture loss. Consequently, the films had lowered mass loss, delayed spoilage, and maintained better sensory quality.

**Fig. 17:**
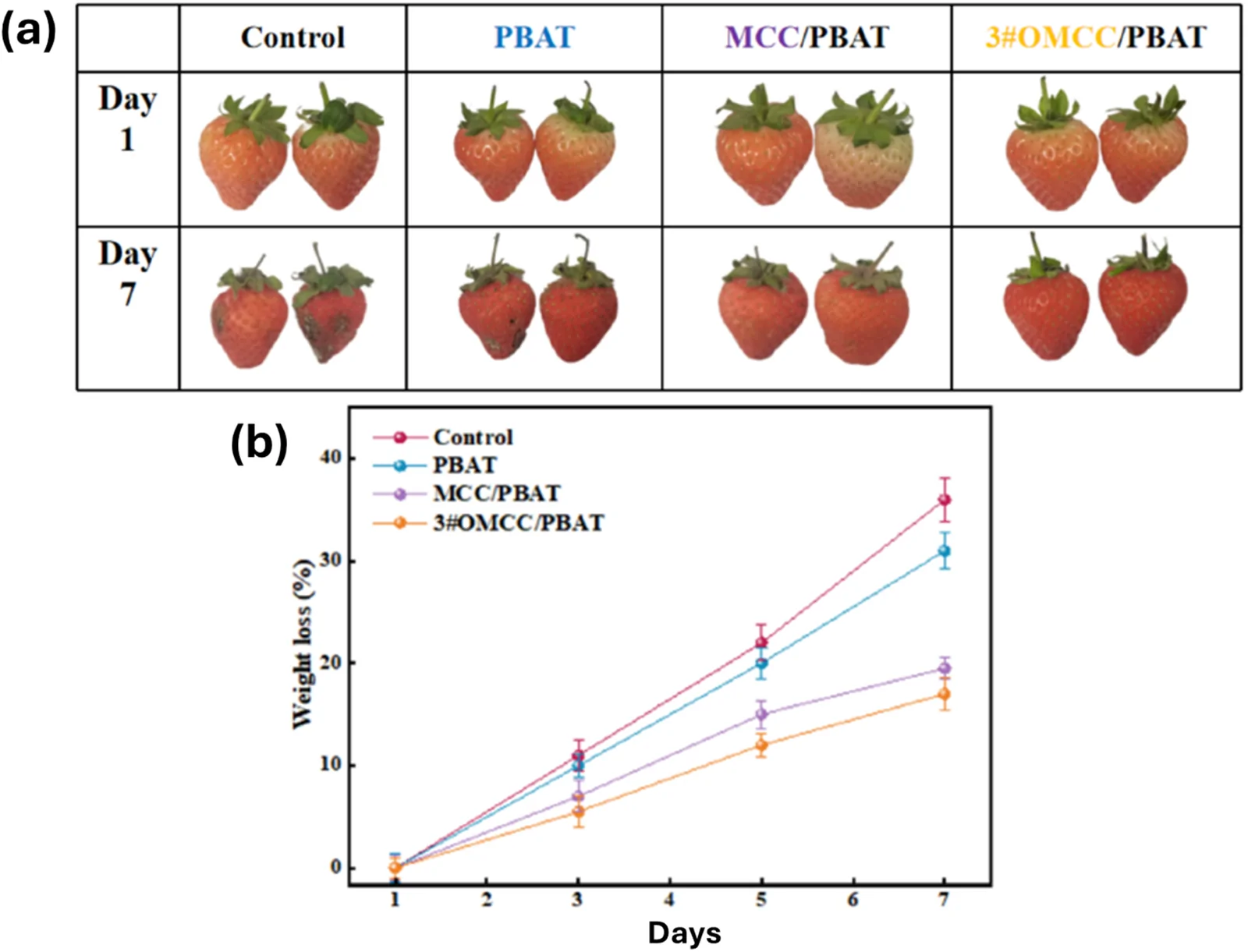
Outcomes of the strawberry preservation test: (a) digital images of the decay progression, and (b) plot of weight loss (%) *vs.* no. days. Reproduced with permission from ref. 130. Copyright 2024. Elsevier Science Ltd.

### 7.5 Photo-stability application

Introducing UV-absorbing additives into biodegradable PBAT films enhances their UV resistance and extends their lifetime in outdoor applications. Qiao *et al.*^131^ developed anti-UV PBAT/UVA films using tropolone-based UV absorbers. The films effectively blocked harmful UV radiation due to significant UV absorption at approximately 238 and 320 nm. PBAT films containing UVA-6C exhibited the maximum absorbance (∼1.26), which was ∼23.8% higher than neat PBAT. This UV-absorbing additives reduce photodegradation by limiting UV-induced chain scission and oxidation reactions. The UV-modified films showed improved durability, with service life extended by up to 240 h under accelerated weathering conditions.

These UV resistant films are useful for agricultural mulch films and outdoor packaging applications, where prolonged sunlight exposure can rapidly degrade the material. The UV-modified films also improved thermal regulation and increased crop yield by up to 7.8%. Their high-molecular-weight UV absorbers showed low migration and good long-term stability, ensuring sustained performance.

The above applications examples demonstrate that the required property balance depends on the intended function of the biodegradable material. For additive manufacturing, increased stiffness alone is insufficient, as the material must also maintain melt flow, interlayer adhesion, and toughness. Hence, the reduction in TS caused by fibre reinforcement required processing modifications such as increased overlap and heat treatment. In contrast, self-cleaning and antimicrobial applications rely primarily on surface functionality, where ZnO incorporation provided photocatalytic and antibacterial activity without requiring the same mechanical property balance as structural applications. For food preservation, moisture retention and barrier performance are more critical than maximizing mechanical strength, as reflected by the lower weight loss obtained with OMCC/PBAT films. Similarly, UV-resistant PBAT films prioritize optical absorption and resistance to photodegradation rather than mechanical reinforcement alone. These examples show that a property change that is beneficial for one application may have limited relevance to another; therefore, material composition, morphology, interfacial interactions, and processing conditions must be selected according to the specific functional requirements rather than to maximize a single property.

## 8 Conclusions and future outlook

This review offers a detailed overview of the structure–property relationships in biodegradable polyester blends. Recent progress in improving the performance of biodegradable polyester blends and composites have been presented and discussed. The reviewed studies show that blend performance is not only related to composition, but is governed by the combined effects of thermodynamic compatibility, phase morphology, interfacial interactions, crystallinity, and processing conditions. Since most biodegradable polyester blends are thermodynamically immiscible, the resulting domain size, phase distribution, interfacial area, and phase connectivity become important factors in determining their final properties. Controlling the interface between the phases is essential for achieving balanced properties as most biodegradable polyesters are thermodynamically immiscible. Reactive compatibilizers, fibres, and nanofillers/structures have been used to improve blend morphology and interfacial adhesion. Morphology refinement and stronger interfacial interactions generally favour stress transfer and mechanical performance, while the resulting changes in phase continuity, crystallinity, and diffusion pathways also influence rheological and barrier behaviour. However, these effects are not universally beneficial, as excessive compatibilization, crosslinking, filler loading, or processing can restrict chain mobility, promote degradation, or adversely affect processability. Biodegradable polyester blends can also be designed with added functionalities to expand their use in packaging, agriculture, biomedical materials and 3D printing.

Processing represents an additional level of control over these structure–property relationships. Changes in temperature, shear, mixing history, and processing route can alter phase dispersion, interfacial area, crystallization, molecular structure, and orientation. An optimum processing window is therefore required rather than simply increasing temperature or shear, since morphology refinement and interfacial reactions must be balanced against polymer degradation and processing stability. Similarly, the effects of compatibilizers, chain extenders, and fillers on biodegradation are not linear. Changes in crystallinity, phase morphology, interfacial structure, and polymer accessibility can either accelerate or slow degradation depending on the blend composition and modification strategy. Thus, improving one property does not necessarily improve the overall performance of a biodegradable blend, and the major challenge remains achieving a balance between mechanical, barrier, rheological, processing, and biodegradation requirements.

Interfacial engineering with computational modelling could assist in predicting the performance and development of biodegradable polyester blends. Molecular simulations can act as a computational microscope to study blend interfaces. These simulations can replicate and deduce interfacial interactions and microstructures which are often difficult to examine experimentally. Machine learning (ML) can also help develop force fields for new biopolymers and additives. It can predict material properties and identify promising blend compositions before experimental testing. Combining simulations and ML with experimental studies could help establish clear relationships between blend microstructures, processing, and final material properties. Still, these computational approaches are still not widely explored for biodegradable polymer blends. Their wider use could reduce experimental effort and support faster and more informed biopolymer blend design.

Compatibilizers and additives that work well at the laboratory scale may behave differently at industrial-scale processes. Changes in processing conditions can affect compatibilizers and additive dispersion, interfacial reactions and overall performance. They can also add to material and processing costs. Industrial scale-up should consider the practical feasibility of compatibilizers and additives alongside their ability to improve material properties. It is equally important to ensure that these additives do not reduce the biodegradability of the blends.

Many emerging applications, including active and intelligent packaging, flexible electronics, sensors, responsive labels, and fire retardants have been reported using neat biopolymers. However, their applications are often limited by the poor overall performance and processing limitations of individual biopolymers. Biopolymer blending provides an opportunity to combine the advantages of different biopolymers and overcome some of these limitations. Better control over the structure and interactions within biodegradable polyester blends could help achieve the balanced properties needed for more demanding applications. Further, the multiphase structure of blends may also provide opportunities to control the location and release of antioxidants, antimicrobial agents, and other active compounds. Improving the properties and compatibility of biopolymer blends could support the incorporation of conductive polymers and biopolyelectrolytes.

Responsive materials provide another promising direction. Colour-changing components could be incorporated into biopolymer blends to indicate changes in pH, temperature, gases, or metabolites associated with food deterioration. Phase-change materials could also be integrated to develop indicators for monitoring temperature exposure during transportation and storage. In these systems, controlling the blend morphology and the location of the functional component could provide additional ways to tune its response and stability.

Future research should therefore consider structure–property enhancement not only as a means of improving the conventional performance of biopolymer blends, but also as a pathway towards new applications. By combining the complementary properties of different biopolymers with active, conductive, or stimuli-responsive components, blends could overcome some of the limitations associated with neat biopolymers and provide more versatile platforms for active packaging, intelligent packaging, flexible sensing, and other multifunctional applications. However, these additional functions should be introduced without compromising the biodegradability, safety, processability, and economic viability of the resulting materials.

## Author contributions

Vaishnu Suresh Kumar: conceptualization, data curation, formal analysis, visualization, writing – original draft, writing – review and editing; Sarbani Ghosh: resources, validation, supervision; Mehran Ghasemlou: formal analysis, validation, writing – review and editing, supervision; Banasri Roy: resources, validation, supervision; Benu Adhikari: validation, supervision; Mohit Garg: conceptualization, formal analysis, resources, writing – review and editing, supervision; Fugen Daver: conceptualization, formal analysis, resource, writing – review and editing, supervision.

## Conflicts of interest

The authors declare no competing financial interest.

## Abbreviations

ABS: Acrylonitrile-butadiene-styrene
ADR: Poly(styrene-acrylic-*co*-methacrylate glycidyl)
CNF: Cellulose nanofiber
CS: Chitosan
DCP: Dicumyl peroxide
EAB: Elongation at break
EM: Elastic modulus
EOS: The equation of state
EPL: ε-Poly-l-lysine
ESO3-ECD: Epoxidized soybean oil-based compatibilizer
FFF: Fused filament fabrication
GMA: Glycidyl methacrylate
HDT: Heat deflection temperature
HT: Heat treatment
IL-*g*-POSS: Ionic liquid-grafted polyhedral oligomeric silsesquioxane
IS: Impact strength
JN: Janus nanosheet
LCST: Lower critical solution temperature
LDH: Layered double hydroxide
LDPE: Low-density polyethylene
MA-*g*-PBAT: Maleic anhydride-grafted PBAT
MFC: Microfibrillated cellulose
PCL: Polycaprolactone
P(MMA-*co*-GMA): Methyl methacrylate-glycidyl methacrylate
PMASQ: Poly(methyl/amino)silsesquioxane
OL: Processing modifications such as overlap
OMCC: Organically modified microcrystalline cellulose
OP: Oxygen permeability
OTR: Oxygen transmission rate
PBAT: Poly(butylene adipate-*co*-terephthalate)
PBS: Poly(butylene succinate)
PBSA: Poly(butylene succinate-*co*-adipate)
PDLA: Poly-d-lactic acid
PGA: Polyglycolide
PHA: Poly(hydroxyalkanoate)
PHB: Poly(3-hydroxybutyrate)
PHBH: Poly(3-hydroxybutyrate-*co*-3-hydroxyhexanoate)
PHBV: Poly(3-hydroxybutyrate-*co*-3-hydroxyvalerate)
PLA: Polylactic acid
PLA-*g*-ECA: Epoxidized cardanol-grafted PLA
PLLA: Poly-l-lactic acid
POSS: Polyhedral oligomeric silsesquioxane
SAOS: Small-amplitude oscillatory shear
SC: Stereocomplex
TMPTA: Trimethylolpropane triacrylate
TPS: Thermoplastic starch
TS: Tensile strength
UV: Ultraviolet
UVA: Ultraviolet absorber
WVTR: Water vapor transmission rate
ZnO: Zinc oxide

## Data Availability

No new data were generated or analysed in this study. All information discussed in this review is available in the cited literature.
